# Comparative de novo transcriptome analysis identifies salinity stress responsive genes and metabolic pathways in sugarcane and its wild relative *Erianthus arundinaceus* [Retzius] Jeswiet

**DOI:** 10.1038/s41598-021-03735-5

**Published:** 2021-12-31

**Authors:** P. Vignesh, C. Mahadevaiah, R. Parimalan, R. Valarmathi, S. Dharshini, Singh Nisha, G. S. Suresha, S. Swathi, H. K. Mahadeva Swamy, V. Sreenivasa, K. Mohanraj, G Hemaprabha, Ram Bakshi, C. Appunu

**Affiliations:** 1 ICAR-Sugarcane Breeding Institute, Coimbatore, India; 2 ICAR-National Bureau of Plant Genetic Resources, New Delhi, India; 3 Queensland Alliance for Agriculture and Food Innovation, University of Queensland, Brisbane, Australia; 4 ICAR-National Institute for Plant Biotechnology, New Delhi, India; 5 Institute for Genomic Diversity, Cornell University, Ithaca, NY 14853 USA

**Keywords:** Genomics, Gene expression, Genomics

## Abstract

*Erianthus arundinaceus* [Retzius] Jeswiet, a wild relative of sugarcane has a high biomass production potential and a reservoir of many genes for superior agronomic traits and tolerance to biotic and abiotic stresses. A comparative physiological, anatomical and root transcriptome analysis were carried out to identify the salt-responsive genes and metabolic pathways associated with salt-tolerant *E. arundinaceus* genotype IND99-907 and salinity-sensitive sugarcane genotype Co 97010. IND99-907 recorded growth of young leaves, higher proline content, higher relative water content, intact root anatomical structures and lower Na^+^/K^+^, Ca^2+^/K^+^ and Mg^2+^/K^+^ ratio as compared to the sugarcane genotype Co 97010. We have generated four de novo transcriptome assemblies between stressed and control root samples of IND99-907 and Co 97010. A total of 649 and 501 differentially expressed genes (FDR<0.01) were identified from the stressed and control libraries of IND99-907 and Co 97010 respectively. Genes and pathways related to early stress-responsive signal transduction, hormone signalling, cytoskeleton organization, cellular membrane stabilization, plasma membrane-bound calcium and proton transport, sodium extrusion, secondary metabolite biosynthesis, cellular transporters related to plasma membrane-bound trafficking, nucleobase transporter, clathrin-mediated endocytosis were highly enriched in IND99-907. Whereas in Co 97010, genes related to late stress-responsive signal transduction, electron transport system, senescence, protein degradation and programmed cell death, transport-related genes associated with cellular respiration and mitochondrial respiratory chain, vesicular trafficking, nitrate transporter and fewer secondary metabolite biosynthetic genes were highly enriched. A total of 27 pathways, 24 biological processes, three molecular functions and one cellular component were significantly enriched (FDR≤ 0.05) in IND99-907 as compared to 20 pathways, two biological processes without any significant molecular function and cellular components in Co 97010, indicates the unique and distinct expression pattern of genes and metabolic pathways in both genotypes. The genomic resources developed from this study is useful for sugarcane crop improvement through development of genic SSR markers and genetic engineering approaches.

## Introduction

Sugarcane is an industry-oriented crop grown in the tropics and sub-tropics in the world. Sugarcane is a potential source of basic material for the manufacturing of sugar, ethanol, bioenergy, and biodegradable products. Traditional sweeteners such as jaggery, khandsari, and brown sugars having immense medicinal value are also produced from sugarcane^1,2^. Globally, sugarcane was cultivated in 28.19 million hectares which produced 2059.74 million tonnes of canes with a productivity of 72.80 t/ha during 2019^3^. In India, sugarcane was cultivated in 5.06 million hectares and 405.42 million tonnes was produced with a productivity of 80.10 tonnes/hectare during 2019^3^. The growth, productivity, and juice quality of sugarcane are affected by abiotic stresses viz*.,* cold, salinity, and drought. Nearly 10% of arable land or 25–30% of irrigated lands were affected by salinity in the world^4^. After the advent of modern agriculture, soil salinity has become a major environmental issue and underground water used for irrigation also contributes to the soil salinity^5^. Sugarcane is highly sensitive to salinity^6^ and affects both biomass accumulation and juice quality parameters^7^. Therefore, the development of saline tolerant varieties, gene pools, and genomic resources are helpful in sugarcane crop improvement through conventional and biotechnological approaches.

The sessile plants are sensing the salinity stress-induced osmotic and ionic stresses through the activation of calcium signalling and salt-overly sensitive pathways for exclusion of sodium^8,9^. Glycosyl inositol phosphoryl ceramide sphingolipids are also involved in the sensing of salinity stress^10^. The salinity stress firstly reduces the water uptake causing the salinity-induced osmotic stress and secondly, increases the concentration of cytotoxic ions which causes ionic stress^5,10^. The plant possesses several mechanisms to tolerate the salinity stress such as (i) accumulation of low molecular weight, water-soluble free state compounds (proline, betaine, water-soluble sugars) which help in the maintenance of osmotic adjustment and plant metabolic activities (ii) selective ion-uptake such as the exclusion of sodium and maintaining higher cytosolic K^+^/Na^+^ (iii) nullifying the effect of ROS by enzymes such as catalase, SOD and APX (iv) salinity-tolerant genes associated with sodium-hydrogen antiporter activities of vacuolar membrane, plasma membrane and, genes related to ROS scavenging enzymes^11^. Besides, many gene networks related to signal transduction, hormone signalling, biosynthesis of secondary metabolites, amino acids and transporters significantly contribute to the salinity tolerance mechanisms in plants^12^. The comparative global gene expression studies are certainly helping to dissect the various genes and metabolic pathways associated with salinity or abiotic stress tolerances in plants^13^.

Sugarcane wild relative *E. arundinaceus* is a potential donor for many genes related to biomass and tolerance to biotic and abiotic stresses^14–16^. Introgression of many genes from *E. arundinaceus* into sugarcane through conventional and biotechnological approaches has improved the agronomic performance of sugarcane varieties^17,18^. We have isolated, characterized, and overexpressed many genes from *E. arundinaceus* such as heat shock protein 70^14,19^, DREB2^20^, glyoxalase gene^15^, α-expansin 1^21^ and chilling tolerant divergence 1 (COLD1) gene^22^, which shows the genetic importance of *E. arundinaceus* in improving the tolerances to biotic and abiotic stresses in sugarcane*.* There were several RNASeq studies in sugarcane for many traits such as agronomic traits, cold stress, sucrose, lignin, red stripe and smut disease except for salinity tolerance^23–28^. Hence, we performed the comparative salt transcriptome studies to identify the salinity stress-responsive genes and metabolic pathways in salt-tolerant *E. arundinaceus* accession IND99-907 collected from saline soils Ernakulum district, Kerala, India^29^ and salt-sensitive genotype Co 97010^30,31^. From this study, we have identified many differentially expressed genes, enriched metabolic pathways and GO terms associated with salt tolerance in salt-tolerant *E. arundinaceus* accession IND99-907. Our studies showed the enrichment of 27 pathways, 24 biological processes, three molecular functions and one cellular component in IND99-907 as compared to 20 pathways, two biological processes without any significant molecular function and cellular components in Co 97010 (FDR ≤ 0.05), which specifies the unique and distinct expression pattern of genes and metabolic pathways regulating the salinity stress in IND99-907 and Co 97010. The genomic resources developed from this study are useful in sugarcane crop improvement through development of genic markers and advanced biotechnological approaches.

## Results

### Physiological and root anatomical assay

**Figure 1:**
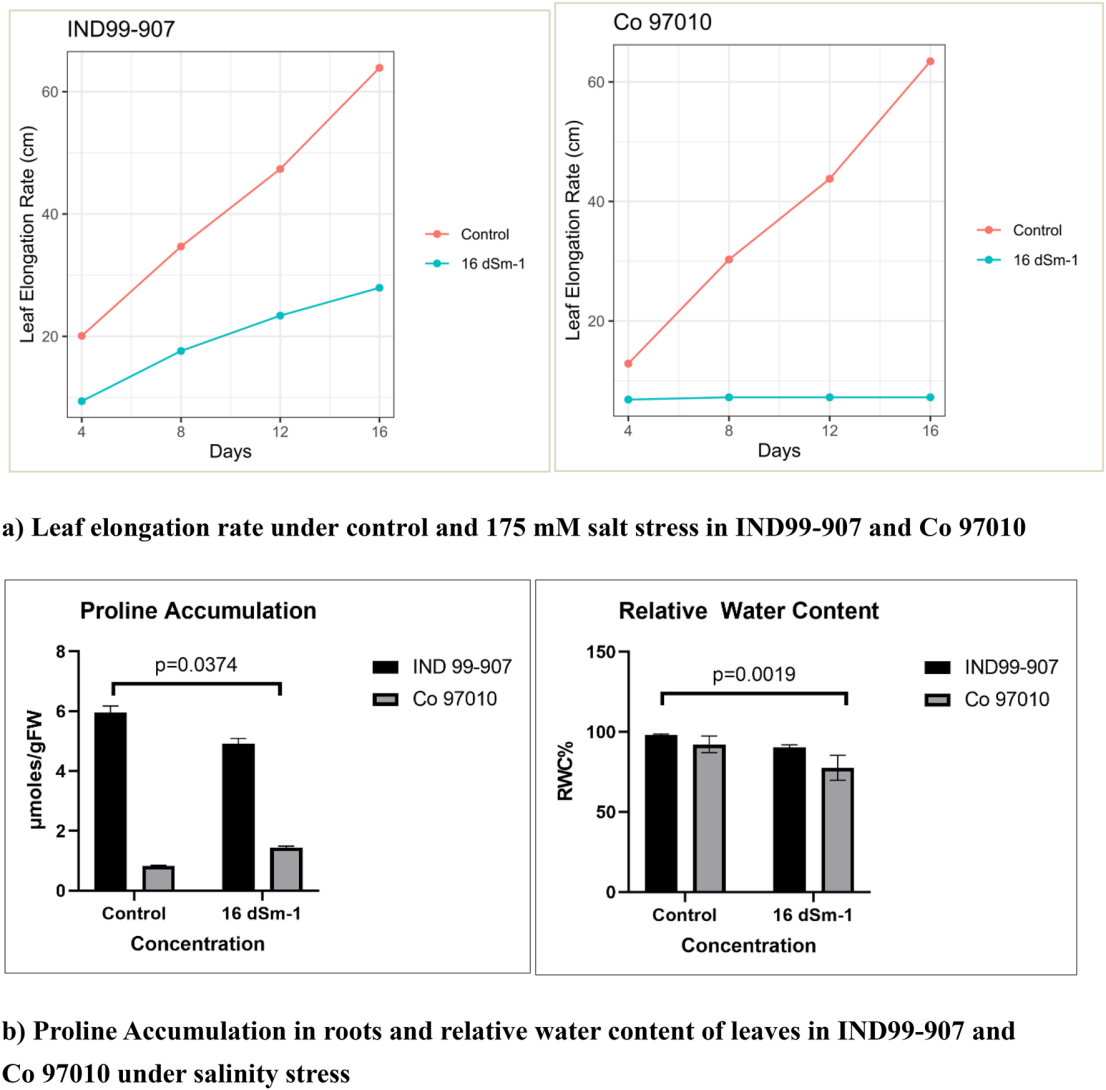
Phenotypic and physiological responses of IND99-907 and Co 97010 under salinity stress (**A**) Leaf elongation rate, (**B**) Proline content of roots and Relative water content in leaves. Error bars indicates the mean ± SD.

Sugarcane wild relative *E. arundinaceus* genotype IND99-907 was collected from the salinity affected area of Padiyattu Kadavu, Ernakulum, India under germplasm exploration programme during 1999 and adopted to high salinity stress with normal plant growth^29^ and a potential source for harnessing the salt stress-responsive genes. On the other hand, the sugarcane genotype Co 97010 is highly sensitive to salinity and has been used as a salt-sensitive standard in many studies^30,31^. The 90 days old plants of IND99-907 and Co 97010 were imposed with salinity stress by irrigating with 175 mM saline water. The leaf elongation rate (LER) was significantly higher in IND99-907 under salinity stress and growth was ceased in Co 97010 (p value = 0.0251) (Fig. 1a). The physiological assay for proline content in roots showed that IND99-907 recorded significantly higher proline content of 8.908 µmoles/gram of root samples as compared to 1.438 µmoles/gram in Co 97010 under salinity stress (p value = 0.0374) (Fig. 1b). The relative water content revealed that IND99-907 has retained the higher water content under the stressed condition as compared to Co 97010 (p value = 0.0019).

**Figure 2:**
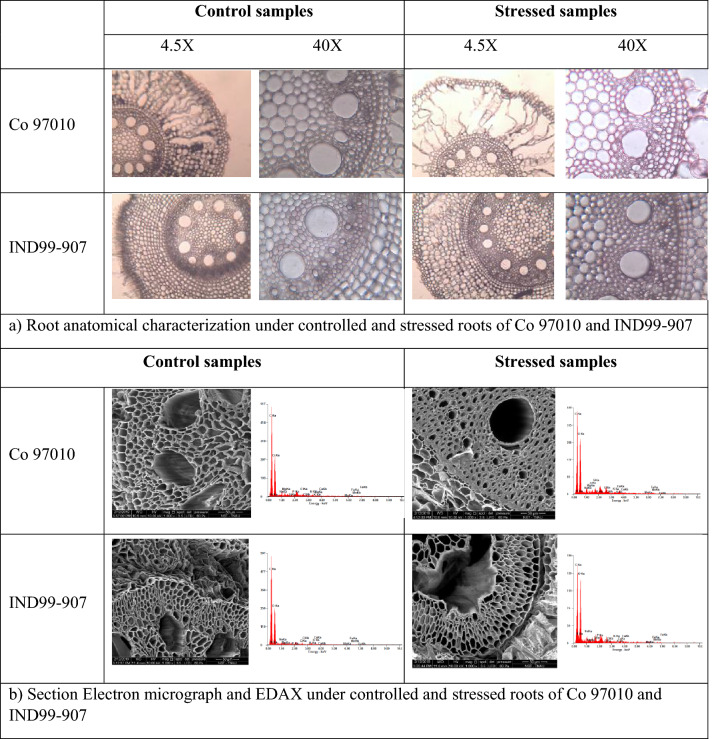
Root anatomical changes in responses of IND99-907 and Co 97010 under salinity stress (**A**) Root anatomical images showing the vacuolization of cortex and thickening of metaxylem vessels in Co 97010 as compared to no significant changes in IND99-907. (**B**) Scanning Electron Micrograph showing thickening of protoxylem with circular xylem vessels in Co 97010 as compared to irregular shaped xylem vessels in IND99-907 and, EDAX analysis showing elemental composition of roots under salinity stress. (**a**) Root anatomical characterization under controlled and stressed roots of Co 97010 and IND99-907. (**b**) Section Electron micrograph and EDAX under controlled and stressed roots of Co 97010 and IND99-907.

The root anatomical studies under light microscope revealed the clear differences in thickening of cells around the metaxylem in IND99-907 and Co 97010. The vacuolization of the cortex was observed in Co 97010 as compared to the normal cortex in IND99-907. The thickening of endodermis and deformed protoxylem vessels were observed in Co 97010 as compared to no significant change in IND99-907 (Fig. 2a). The same results were confirmed in Scanning Electron Microscope which showed cell-wall thickening in protoxylem and thickening with circular xylem vessels in Co 97010. IND99-907 revealed xylem vessels without a significant change in protoxylem and metaxylem (Fig. 2b). Similar changes in the root cellular structure under drought and salinity stress in the tolerant genotypes are attributed to be an adaptive mechanism of resistant genotypes^32,33^.

The elemental composition of stressed and control samples were analyzed by SEM- EDX and ICP-OES methods. The differential accumulation pattern of sodium, calcium, magnesium, and potassium under both control and stressed root samples were observed in IND99-907 and Co 97010. The lowest Na^+^/K^+^ ratio of 0.64 was observed in IND99-907 as compared to 1.50 in Co 97010 by ICP-OES analysis. A similar trend of lowest Na^+^/K^+^ was observed in IND99-907 (2.18) as compared to 7.64 by Co 97010 (Table 1 and Fig. 2b) in SEM–EDX analysis. Similarly, IND99-907 also recorded the lowest Ca^2+^/K^+^ (1.05) and Mg^2+^/K^+^ (0.23) ratio as compared to 4.52 and 0.73 in Co 97010 respectively under salinity stressed condition.

**Table 1:** Estimated Sodium–Potassium ratio by ICP-OES and SEM–EDX method at 175 mM for 15 days in pot culture experiment.

Genotypes	Na^+^/K^+^ ratio		Ca^+^/K^+^ ratio		Mg^+^/K^+^ ratio	
	S	C	S	C	S	C
**(i) ICP-OES analysis**						
Co 97010	7.64	1.88	4.52	2.30	0.79	0.51
IND99-907	2.18	0.75	1.05	0.45	0.23	0.18
**(ii) SEM–EDX analysis**						
Co 97010	1.50	0.80	3.24	1.06	0.33	0.71
IND99-907	0.64	1.03	0.00	0.87	0.18	1.17

### Transcriptome data overview

The comparative transcriptome analysis was performed between stressed and control samples of saline-tolerant *E. arundinaceus* accession IND99-907 and saline-sensitive sugarcane genotype Co 97010 with three biological replications. A total of 305, 244, 264, and 263 million raw reads were obtained from the stressed and control libraries of IND99-907 (marked as 907S and 907C) and Co 97010 (marked as 97010S and 97010C) respectively (Supplementary Table S1). The pre-processing of raw reads by removal of adapter sequences, rRNA sequences, and ambiguous bases with high-quality sequences with a Phred score of ≥ 30 (Q30) resulted in 226, 182, 196, and 198 million clean reads from stressed and control libraries of IND99-907 and Co 97010 respectively. We obtained more than 74% of the high-quality clean reads after pre-processing of the raw data resulting in 802 million clean reads which were good enough to perform the RNASeq analysis.

### De novo assembly of transcripts and unigenes clustering

The high-quality reads of stressed and control libraries of IND99-907 and Co 97010 were assembled into four de novo assemblies for 907C, 907S, 97010C and 97010S using Trinity v2.8.5. The quality assessment and comparison of assemblies were performed using Quast and BUSCO tools^34,35^. (i) The Quast analysis revealed the total number of contigs of four assemblies ranging from 205,717 to 345,745, with the highest contig N_50_ of 1727 bp observed in 907S assembly and the largest contig of 29,724 bp was recorded in 97010S assembly. (ii) BUSCO analysis showed the completeness percentage ranging from 69.8% (907C) to 80% (907S) with a maximum duplication of 42.1% was observed in 97010C assembly. After unigenes clustering, the number of contigs ranged from 179,304 to 242,390 with the highest contig N_50_ of 2102 bp in 907S unigenes assembly. The merging of control and stressed unigene assemblies for each genotype resulted in the identification of 315,611 unigenes with a contig N_50_ of 2071 bp and 426,820 unigenes with N_50_ of 1800 bp in clustered unigene assembly of IND99-907 and Co 97010 respectively (Supplementary Table S1). The high quality clustered assemblies of IND99-907 and Co 97010 were used for transcript quantification and annotation processes.

### Transcript abundance estimation and differential gene expression

**Figure 3:**
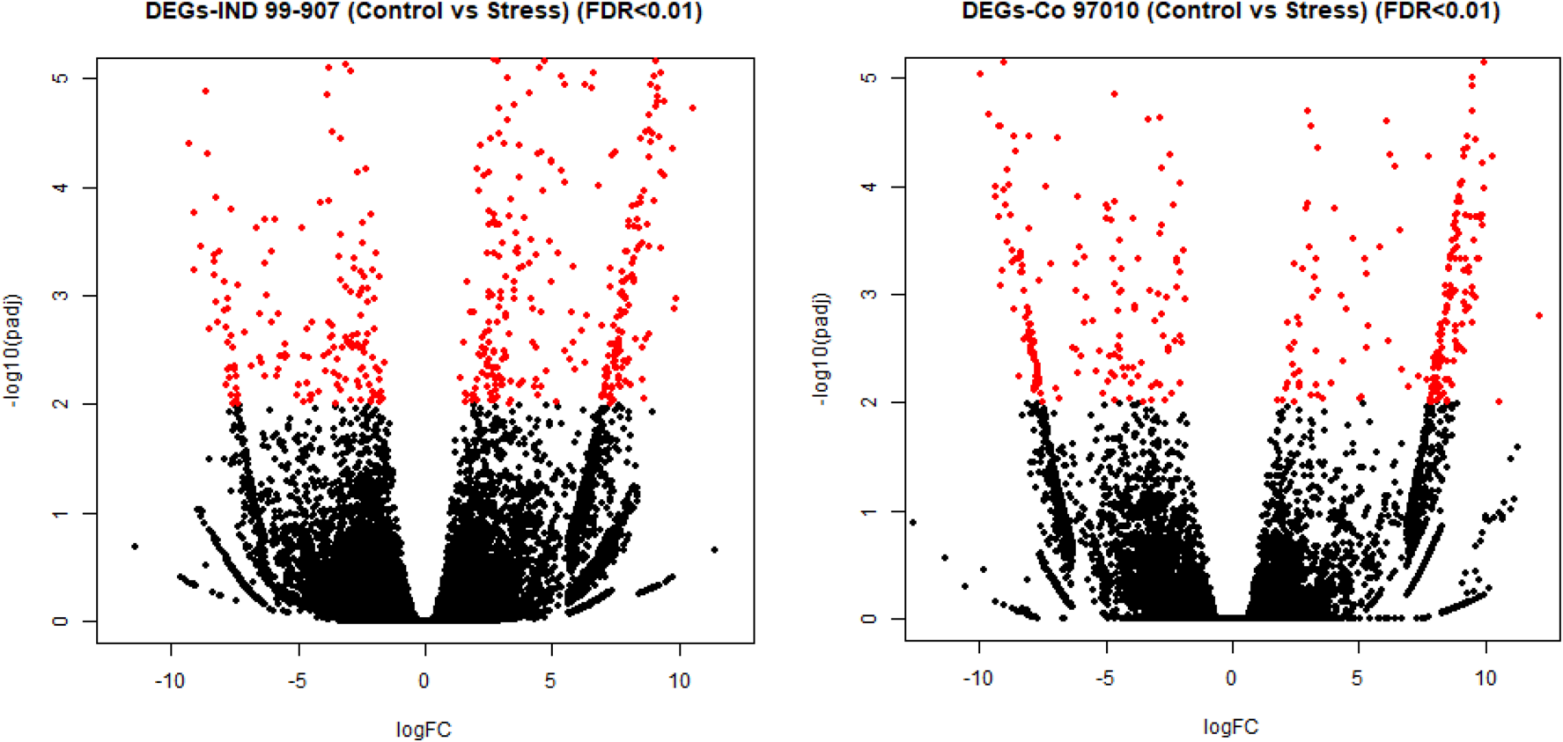
Volcano plots displaying the distribution of DEGs between control vs stress conditions of (**a**) IND99-907 and (**b**) Co 97010 based on FDR < 0.01.

The coding DNA sequences were identified from the clustered assemblies of IND99-907 and Co 97010 by using the TransDecoder tool and the transcript quantification was performed by using RSEM v1.2.25. The transcript counts generated by RSEM were used to perform the differential gene expression (DGE) analysis by using DESeq2 for both IND99-907 and Co 97010 separately. The DEGs were identified based on absolute log_2_FC > 2 for upregulated genes and log_2_FC < -2 for downregulated genes. A total of 7731 DEGs from IND99-907 (Control vs Stress) DEG library and 6159 DEGs from Co 97010 (Control vs Stress) library were obtained at significance level p < 0.05. The FDR corrections were performed through the Benjamini–Hochberg method^36^ to reduce the false positives and, a stringent FDR < 0.01 was used to further reduce the number of DEGs which yielded a total of 649 DEGs in IND99-907 and among them, 425 genes were upregulated and 197 were downregulated. A total of 501 DEGs (FDR<0.01) were identified in Co 97010 and out of them, 283 genes were upregulated and 213 genes were downregulated (Fig. 3). These DEGs (FDR<0.01) identified in IND 99-907 and Co 97010 were used for functional annotation.

### Functional mapping of DEGs

The functional annotation of DEGs (FDR < 0.01) of IND99-907 and Co 97010 were carried out by using tools such as Blast2GO, HMMer & Command-line Blast against NCBI-nr, Swissprot, Pfam, and Uniprot Plant databases. The results against the NCBI-nr database for total DEGs (FDR < 0.01) showed 88.4% and 90.6% annotations for IND99-907 and Co 97010 respectively. Gene Ontology (GO) annotations were obtained for all DEGs. Upregulated and downregulated genes were also annotated separately using Blast2GO for both IND99-907 and Co 97010. In IND99-907, 88.2% of the upregulated and 93.4% of the downregulated genes were blasted with hits respectively and for Co 97010, 91.2% of upregulated and 93.4% of downregulated genes had blast annotations. The top hit species of DEGs were *Sorghum bicolor, Zea mays, Setaria italica,* and *Setaria viridis*. A total of 81 DEGs in IND99-907 had fold change values (log_2_FC) greater than 20, which includes ATP synthase, abscisic stress ripening protein 2, E3 ubiquitin-protein ligase AIRP2, serine/threonine-protein kinase WNK1 and ABC Transporter F (Supplementary Table S2). In total, five stress-related proteins, nine heat-shock proteins (HSPs), 25 transporters, 46 kinases, and 37 transcription factors (TFs) were significantly differentially regulated (FDR < 0.01) under salt stress. Similarly, for Co 97010, only eight DEGs had fold change values > 20 which included three hypothetical proteins, two ribosomal proteins, and two dehydrogenases. Three stress-related DEGs were identified in Co 97010, namely salt stress-induced hydrophobic peptide ESI3 (ESI3), heat stress transcription factor C-2a (HSFC2A) and abscisic stress ripening protein 2 (ASR2), out of which ESI3 and HSFC2A were upregulated and ASR2 was downregulated under stress conditions. Further, a total of six heat shock proteins, 18 transporters, 18 kinases, and 19 TFs were identified in Co 97010. Gene families were identified for both DEGs of IND99-907 and Co 97010, which showed 413 gene families present in 649 DEGs of IND 99-907 and 327 gene families present in 501 DEGs of Co 97010.

### Gene ontology (GO) enrichment

Gene Ontology (GO) annotations for DEGs were categorized into three GO functional classifications as cellular component (CC), molecular function (MF) and biological process (BP)^37^. A total of 78.7% hits in IND99-907 and 78.2% in Co 97010 were mapped using GO analysis. GO enrichment analysis showed significant enrichment (FDR < 0.05) of 17 different processes for 649 DEGs in IND99-907. The significantly enriched stress-related processes (FDR < 0.05) were given in Supplementary Table S3, S4 & Supplementary Figure S1A. Similarly, the GO enrichment of MF and CC were also studied to identify the significant enrichments at FDR ≤ 0.05 correspondingly which showed two molecular functions namely transporter (GO:0005215) and transferase (GO:0016740) activity (Supplementary Table S3 & Supplementary Figure S1B) and one cellular component namely vacuole (GO:0005773) (Supplementary Figure S1C) under salinity stress in IND99-907. Whereas in Co 97010, two biological processes namely metabolite and energy precursor generation (GO:0006091) and response to stress (GO:0006950) were significantly enriched among 501 DEGs at FDR ≤ 0.05 (Supplementary Table S4) and no significant enrichment at FDR ≤ 0.05 was observed for cellular components and molecular functions.

### KEGG annotation and enrichment

KEGG annotations and Enzyme Code (EC) mapping were performed using Blast2GO. A total of 79 different KEGG pathway hits were obtained for 649 DEGs in IND99-907 and 67 KEGG pathway hits were obtained for 501 DEGs in Co 97010 with both of them having major hits in Glycolysis/Gluconeogenesis (ko00010), Pyruvate metabolism (ko00620), Galactose metabolism (ko00052) and Starch and Sucrose metabolism (ko00500). Further, KEGG enrichment showed a total of 27 statistically significant (FDR < 0.05) enriched KEGG pathways in IND99-907 (Supplementary Table S5) including Endocytosis (ko04144), Lysine degradation (ko00310), Plant hormone signal transduction (ko04075), Oxidative phosphorylation (ko00190) and MAPK signalling pathway (ko04016). In contrast, DEGs of Co 97010 showed 20 significantly enriched KEGG pathways (FDR < 0.05) (Supplementary Table S6) such as Oxidative phosphorylation (ko00190), Metabolic pathways (ko01100), and Photosynthesis (ko00195).

### Endocytosis

Endocytosis, an important pathway for the intracellular vesicle trafficking system was significantly enriched (FDR = 3.18E-09) in total DEGs of IND99-907 under salinity stress. A total of nineteen transcripts were differentially regulated and among them, seven were upregulated and twelve were downregulated. Among the upregulated gene hits in the pathway, clathrin interactor epsin-2 showed the highest upregulation (log_2_FC = 20.31). Other genes and their fold change values were ras-related RabE1d protein (log_2_FC = 8.77), ArfGAP (log_2_FC = 7.72), phospholipase D (log_2_FC = 7.990 and HSP70 (log_2_FC = 3.49). It was observed that the same pathway was significantly enriched (FDR = 0.001157) in the DEGs Co 97010 with two of the proteins ADP-ribosylation factor 1 (log_2_FC = − 9.21) and vacuolar protein sorting associated protein (log_2_FC = − 3.49) being highly downregulated under salinity stress (Supplementary Figure S2).

### Oxidative phosphorylation

Oxidative phosphorylation is a pathway associated with the formation of ATP through phosphorylation of NADH or FADH_2_ by electron transport chain. This pathway was significantly enriched at FDR = 0.005099 in DEGs of IND 99-907 with a total of nine transcripts involving in the pathway. The highest upregulation was observed in the protein ATP synthase (log_2_FC = 22.66) which catalyses the formation of ATP. Several ATPase hits were observed in the pathway like calcium-transporting, plasma membrane, V-type proton ATPases and the highest upregulated was observed in ATPase subunit 6 (log_2_FC = 21.09). The same pathway was also significantly enriched (FDR = 7.45E−16) in the DEGs of Co 97010 with a total of twenty transcripts involving in the pathway including dehydrogenase, cytochrome c oxidase, and ATP synthase. The highest down regulation was observed in cytochrome c oxidase (COX) (log2FC = − 26.86) followed by ATP synthase CF1 beta subunit (log2FC = − 25.00) (Supplementary Figure S3).

### qRT-PCR validation of transcriptome data

A total of 43 DEGs representing the various metabolic pathways in both IND 99-907 and Co 97010 were chosen for qRT-PCR validation. The details of the genes selected for validation and their primers are given in Table 2. Dehydrin DHNs are a group of LEA proteins involved in membrane stabilization during abiotic stresses^38^ was upregulated in both IND99-907 and Co 97010 and the same was reconfirmed in qRT-PCR. NADH dependent malic enzyme upregulated in response to abscisic acid signalling and drought^39^ was upregulated in IND99-907 and downregulated in Co 97010. Dirigent-like protein associated with lignin biosynthesis and cellular stabilization^40^ was upregulated in IND99-907 and nil expression was observed in Co 97010. Katanin is a microtubule^41^ and its regulating gene Katanin p80 WD40 repeat was upregulated in IND99-907 and nil expression was observed in Co 97010. Total of 43 genes were validated using qRT-PCR and correlation of 0.53 was observed between RNAseq and quantitative gene expression by qRT-PCR.

**Table 2:** Validation of candidate genes through qRT-PCR.

Pathway	Putative functions	Gene name	Protein name	Unigene ID		IND99-907		Co 97010	
				IND99-907	Co 97010	qRT-PCR	RNA Seq	qRT-PCR	RNA Seq
Membrane Stabilization	LEA proteins involved in membrane stabilization^38^	DHN1	Dehydrin DHN1	ERI-C_DN10750_ c0_g1_i1.p1	SUG-S_DN859_ c0_g1_i3.p1	5.8	5.81	8.12	6.28
Membrane Stabilization	lignin biosynthesis and cellular stabilization^40^	DIR1	Dirigent-like protein	ERI-S_DN657_ c0_g1_i9.p1		2.51	3.14	− 0.76	–
Membrane Stabilization	Microtubules organization^41^	KTN80.4	Katanin p80 WD40 repeat-containing subunit B1	ERI-S_DN8743_ c0_g1_i22.p1		10.25	3.72	0.26	–
Membrane-bound trafficking	clathrin-mediated endocytosis^92^	EPSIN2	Clathrin interactor EPSIN 2	ERI-S_DN1711_ c0_g1_i13.p1		2.55	20.3	− 0.59	–
Membrane-bound trafficking	cellular cargo located on golgi bodies and plasma membrane^95^	RABE1D	Ras-related protein RABE1d-like	ERI-S_DN1918_ c0_g1_i19.p2		3.14	8.77	− 0.47	–
Metabolic Process	Carbohydrate metabolism^118^	PFK6-like	ATP-dependent 6-phosphofructokinase 6-like	ERI-S_DN1551_ c0_g1_i5.p1	SUG-C_DN1191_ c0_g1_i35.p1	− 1.05	− 2.22	− 0.46	2.46
Metabolic Process	Carbohydrate metabolism^119^	SUS2	Sucrose synthase 2	ERI-C_DN1377_ c0_g1_i34.p3		− 1.34	− 11.04	− 0.2	–
Metabolic Process	Carbohydrate metabolism^71^	DLAT2	Dihydrolipoyllysine-residue acetyltransferase component 2 of pyruvate dehydrogenase complex mitochondrial	ERI-C_DN16417_ c0_g1_i16.p1		1.58	20.43	0.03	–
Metabolic Process	secondary metabolites^72^	GAD1	glutamate decarboxylase	ERI-S_DN184_ c0_g1_i30.p1	SUG-S_DN212_ c0_g2_i1.p1	11.4	10.87	1.49	1.45
Metabolic Process	secondary metabolites^75^	LKR	Lysine-ketoglutarate reductase/saccharopine dehydrogenase1	ERI-S_DN817_ c0_g1_i119.p1		5.89	6.19	0.94	–
Metabolic Process	Sterol synthesis^120^	SMO2-1	methylsterol monooxygenase 1–1 isoform X2	ERI-S_DN4522_ c0_g1_i1.p1		5.96	5.77	0.02	–
Metabolic Process	Sterol synthesis	STE1	Delta(7)-sterol-C5(6)-desaturase 1	–	SUG-S_DN756_ c0_g1_i20.p2	− 0.58	–	4.32	8.99
Metabolic Process	Monoterpenoid/secondary metabolite biosynthesis	7DGT	7-deoxyloganetin glucosyltransferase	ERI-S_DN2625_ c1_g2_i2.p2	SUG-C_DN47939_ c0_g1_i1.p3	1.25	2.74	− 1.81	-3.70
Metabolic Process	arabinogalactan‑protein biosynthesis—cell wall expansion	GLCAT14A	beta-glucuronosyltransferase GlcAT14A-like	ERI-S_DN7775_ c0_g1_i3.p2		1.69	5.24	− 0.94	–
Metabolic Process	Hypusine biosynthesis	DHS	deoxyhypusine synthase	ERI-S_DN2053_ c0_g1_i2.p2		4.7	20.44	− 0.48	–
Metabolic Process	Cell wall organization	GDPDL3	Glycerophosphodiester phosphodiesterase GDPDL3	ERI-C_DN1124_ c0_g1_i5.p1		2.31	7.42	0.91	–
Metabolic Process	Osmoprotectant	HSP23.6	23.6 kDa heat shock protein, mitochondrial	ERI-S_DN974_ c0_g1_i3.p2	SUG-S_DN78847_ c0_g1_i1.p1	1.2	3.31	5.03	6.08
Metabolic Process	Secondary metabolites^74^	AKR1B1	aldose reductase	ERI-S_DN5048_ c1_g1_i18.p1	SUG-S_DN8120_ c0_g1_i6.p1	4.59	4.31	3.52	2.44
Metabolic Process	Secondary metabolites- proanthocyanidin biosynthesis	BAN	Anthocyanidin reductase	–	SUG-S_DN242_ c0_g1_i1.p1	− 0.26	–	1.5	3.28
Metabolic Process	Osmo-protectant	LEA3	late embryogenesis abundant protein 3	ERI-S_DN1898_ c0_g1_i22.p1	SUG-C_DN6306_ c0_g1_i16.p2	7	9.44	2.86	3.5
Signal Transduction	Phospholipid signalling	PLDZETA1	phospholipase D zeta 1 isoform X1	ERI-S_DN2802_ c0_g1_i47.p1	SUG-C_DN8702_ c0_g1_i47.p2	2.23	8	4.16	8.02
Signal Transduction	Signalling^93^	CPK15	Calcium-dependent protein kinase 15	ERI-S_DN497_ c0_g1_i36.p1		23.71	20.22	− 0.12	–
Signal Transduction	Abscisic acid mediated signalling^94^	SIP1	stress inducible protein coi6.1	ERI-S_DN921_ c0_g1_i3.p2		8.75	8.84	− 0.75	–
Signal Transduction	Abscisic acid biosynthesis	NCED3	9-cis-epoxycarotenoid dioxygenase 1, chloroplastic	ERI-S_DN1264_ c0_g2_i5.p1	SUG-S_DN43107_ c0_g1_i6.p1	3.68	3.06	1.61	3.35
Signal Transduction	Abscisic acid mediated signalling	PLDalpha1	phospholipase D alpha 1	ERI-C_DN805_ c0_g1_i2.p1		1.69	7.74	− 0.06	0
Signal Transduction	transcriptional activator	ARF7	Auxin response factor 7	ERI-S_DN1671_ c0_g1_i6.p1	SUG-C_DN1108_ c0_g1_i50.p1	9.62	20.53	2.84	7.34
Signal Transduction	abscisic acid mediated signalling^39^	NADP-ME1	NADP-dependent malic enzyme	ERI-S_DN965_ c0_g1_i69.p1	SUG-C_DN4380_ c0_g1_i1.p1	5.75	20.47	− 1.13	− 4.73
Signal Transduction	Abscisic acid mediated signalling	AIRP2	E3 ubiquitin-protein ligase AIRP2-like	ERI-S_DN2132_ c0_g1_i8.p1		2.98	21.09	− 0.3	–
Signal Transduction	Signal Transduction	Osmo-protectant	Embryogenesis transmembrane protein-like	ERI-S_DN415_ c0_g1_i6.p3	SUG-C_DN1841_ c0_g1_i2.p1	12.61	7.14	− 3.3	− 1.98
Signal Transduction	benzothiadiazole (BTH)- mediated cell death-Defence mechanism	SNL6	Cinnamoyl-CoA reductase-like SNL6	ERI-C_DN1176_ c1_g1_i2.p1	SUG-C_DN1270_ c0_g1_i10.p1	− 1.08	− 2.43	11.89	8.83
Solute Transport	calcium fluxes^82^	ACA10	calcium-transporting ATPase 10, plasma membrane-type	ERI-S_DN107_ c0_g1_i2.p1		1.39	20.56	0.52	–
Solute Transport	active proton transport and sodium extrusion^84^	PMA1	plasma membrane ATPase 1	ERI-S_DN2505_ c0_g1_i2.p1	SUG-S_DN3426_ c0_g1_i35.p2	14.48	9.15	1.92	3.99
Transcriptional Regulation	splicing of pri-mRNA	matK	maturase K	-	SUG-C_DN8_ c1_g1_i7.p1	0.04	0	4.22	13.99
Transcriptional Regulation	Transcriptional factor-Flowering pathway^53^	MADS26	MADS-box transcription factor 26	ERI-S_DN1719_ c0_g1_i2.p1		6.98	10.3	0.34	0
Transcriptional Regulation	Transcriptional factor-drought and salt tolerance	NAC74	NAC domain-containing protein 74	ERI-S_DN392_ c0_g1_i7.p1		3.44	20.26	0.12	0
Transcriptional Regulation	nuclear import receptor for serine-arginine rich proteins	MOS14	Transportin MOS14	ERI-S_DN7275_ c0_g1_i16.p1		5.96	7.98	0.42	0
Transferase Activity	Metalic homeostasis	naat-A	Nicotianamine aminotransferase A	ERI-S_DN110_ c0_g1_i3.p1		4.2	3.18	0.02	0
Transporter Activity	Transport^91^	CAT2	Cationic amino acid transporter 2, vacuolar	ERI-C_DN2868_ c0_g1_i3.p3		23.99	20.48	− 0.31	0
Transporter Activity	secondary metabolites^73^	KCS11	3-ketoacyl-CoA synthase 11-like	ERI-S_DN506_ c0_g1_i8.p1		3.02	10.85	− 0.39	0
Transporter Activity	Sugar transporter	CSTLP3	CMP-sialic acid transporter 3	ERI-S_DN14571_ c0_g2_i1.p1		5.77	7.19	− 0.18	0
Transporter Activity	iron transporter	IRT2	fe(2 +) transport protein 2	ERI-C_DN1057_ c0_g1_i4.p1	SUG-S_DN5534_ c0_g1_i2.p1	− 1.1	− 4.74	− 6.94	− 2.05
Transporter Activity	Efflux of glucose	GLT	probable plastidic glucose transporter 2	ERI-S_DN3048_ c0_g1_i50.p1		1.13	3.92	0.15	0

## Discussion

Sugarcane is a glycophyte and grows poorly in saline soils^6^. Salinity stress is a main restrictive feature and significantly affect the growth and biomass accumulation^42,43^. Sugarcane wild relative *Erianthus arundinaceus* is a potential crop for the production of high biomass and reservoir of many genes related to agronomic, biotic and abiotic stresses. We have isolated and characterized many genes from *E. arundinaceus*^14,15,19–22^ and many of genes are still unexplored. Besides, transcriptome studies were reported for many traits such as cold transcriptome^23^, sucrose^24^, agronomic traits^25^, lignin^28^, red stripe disease^26^, smut disease^27^ except for salinity stress. Hence, we performed the comparative transcriptome analysis to identify the total salt-responsive genes and metabolic pathways associated with salt tolerance in *E. arundinaceus* genotype IND99-907 and salt-sensitive sugarcane genotype Co 97010.

Physiological analyses like leaf elongation rate, relative water content, proline accumulation, root anatomical structure and Na^+^/K^+^ ratio revealed the salinity tolerance level of IND99-907. The growth of young leaves was ceased in Co 97010 under salinity stress as compared to growing young leaves in IND99-907. The growth of young leaves in tolerant genotypes was also reported in other crops such as maize^44^ and sorghum^45^. The accumulation of proline was very high in IND99-907 under salinity stress as compared to Co 97010. Proline acts as an osmolyte and protects the sub-cellular structures by scavenging of free-radicals and optimizing the osmotic potential during abiotic stresses^46^. IND99-907 maintained the higher level of relative water content under salinity stress as compared to Co 97010 and this is in confirmation with previous reports maintaining the higher relative water content in tolerant genotypes^19^.

Soil-salinity exhibits higher osmotic potential in soil and creates physiological drought for plants^5^. The root protoxylem and metaxylem thickness also change during exposure to abiotic stresses^47^. In our study, Co 97010 showed a clear lignification of cell wall around the metaxylem constituting to the cell-wall thickening, vacuolization of the cortex and damaged protoxylem in Co 97010 under salinity stress as compared to normal root structures in IND99-907. The higher concentration of sodium in tissues interfere with the metabolic processes and causes cellular death^5^. The tolerant genotypes minimize the cytotoxic effect of sodium and other cations through ionic exclusion, compartmentalization of ions and maintenance of turgor potential through increased uptake of K^+^ ions^5^. In our study, both EDAX and ICP-OES analysis showed the lower Na^+^/K^+^ ratio in IND99-907 and the lower Na^+^/K^+^ ratio is essential for the maintenance of optimal physiological activities such as stomatal movement, photosynthesis and transpiration under salinity stress^48^.

The genome-wide expression of genes for agronomic traits, biotic and abiotic stresses are studied through NGS based transcriptome sequencing in many crops including non-model crops^13^. The comparative transcriptome analysis was performed to identify the salt-responsive genes and metabolic pathways associated with the salt-tolerant *E. arundinaceus* genotype IND99-907 and sugarcane salt-sensitive genotype Co 97010. We have generated 107.71 GB of raw data and 80.28 GB of clean data from IND99-907 and Co 97010. We assembled the high-quality clean reads (Q30) into four different assemblies with the N_50_ values of 1727 bp, 1506 bp, 1389 bp, and 1359 bp respectively for 907S, 907C, 97010S and 97010C, which is found to be superior or on par with the previous transcriptome studies in sugarcane such as cold transcriptome^23–28^. In our study, we identified 315,611 and 426,820 unigenes for both IND99-907 and Co 97010 respectively. The stringent computational protocol such as p-value adjustment or false discovery rate (FDR ≤ 0.01) based on the Benjamini–Hochberg method was adopted to control the false positives during differential gene expression analysis^36^. A total of 649 and 501 DEGs (FDR < 0.01) were identified from IND99-907 and Co 97010 respectively reveals the salt-responsive genes and metabolic pathways associated with salt tolerance in *E. arundinaceus* and sugarcane.

Transcription factors are the major driving factors in the regulation of gene expression and downstream activation of the cascade of metabolic pathways in biotic and abiotic stresses^49^. In our study, DREB1C was upregulated (log_2_FC = 8.70) in IND99-907 and, its overexpression in tobacco and *Arabidopsis* has enhanced the tolerance levels to salt and freezing stresses^50^. The DREB1H was upregulated (log_2_FC = 8.52) in IND99-907 and it is in confirmation with other crops for cold and salt stresses^51^. The overexpression of G-box-binding factor 3-like conferred the tolerance to salt stress in *Arabidopsis*^52^. MADS26, associated with meristem response and growth^53^ and it was also upregulated (log_2_FC = 10.30) in IND99-907. MYB related transcriptional factors regulating abiotic stress tolerance such as MYB-308-like, MYB4-like, MYBAS2^54–57^ were all upregulated in IND99-907.

The GO enrichment analysis is a statistical approach to identify the enriched or over-represented and depleted group of genes and to compare the functional profile of DEGs and metabolic pathways^79^. In our study, ‘GO term: Response to stress’ is significantly enriched in both IND99-907 (FDR = 1.60E-07) and Co 97010 (FDR = 0.031). It is in confirmation with the previous report of GO term: response to stress under salinity stress^80^. In salt-tolerant IND99-907, most of the enriched genes are associated with regulation of cell wall and cytoskeleton reorganization such as (i) Actin (log_2_FC = 21.48) which dynamically regulates the membrane permeability and cytoskeleton organization^81^; (ii) Plasma membrane-type calcium-transporting ATPase 10 (log_2_FC = 20.56) regulating the calcium fluxes, plant immunity and developmental process^82^; (iii) cellulose synthase A7 regulating the secondary cell wall synthesis^83^ (iv) plasma membrane ATPase 1 (log_2_FC = 9.15) regulating the active proton transport and sodium extrusion under salt-stress and its upregulation has been observed to boost the salt tolerance in transgenic events^84^. We have validated two DEGs namely Plasma membrane-type calcium-transporting ATPase 10 and plasma membrane ATPase 1 through qRT-PCR in IND99-907. Whereas, the GO enriched genes in salt-sensitive Co 97010 are majorly representing the metabolic pathways viz*.,* (i) cytochrome c maturase subunit C (log_2_FC = 12.14) associated with electron transport system^85^, (ii) 2,3-bisphosphoglycerate-independent phosphoglycerate mutase-like (log_2_FC = 11.00) associated with glycolysis^86^; (iii) cysteine proteinase 1 (log_2_FC = 9.52) associated with senescence, protein degradation and apoptosis^87^ and (iv) Histidine kinase 4 associated with cytokinin signalling^88^. This has shown that genes and metabolic pathways were uniquely and differentially enriched in salt-tolerant IND99-907 and salt-sensitive Co 97010 for GO term: Response to stress.

The ‘GO term: Transport’ is significantly enriched in IND99-907 (FDR = 0.01) as compared to non-significant enrichment (FDR = 0.06) in Co 97010. Membrane transport plays a major role in plant systems and endomembrane trafficking for adjusting to the changing environmental stresses^89^. Most of the enriched genes in IND99-907 were related to membrane-bound trafficking and stress-related genes. Genes such as (i) nucleobase-ascorbate transporter 3 (log_2_FC = 20.48), also known as nucleobase:cation-symporter 2 associated with nucleobase transportation^90^ (ii) vacuolar cationic amino acid transporter 2 (log_2_FC = 20.48), which is located on tonoplasts and regulate the soluble amino acid concentration in the cytoplasm^91^ (iii) clathrin interactor EPSIN-2 (log_2_FC = 20.30) associated with clathrin-mediated endocytosis^92^; (iv) Calcium-dependent protein kinase 15 (log_2_FC = 20.22) interacts with WRKY transcriptional factors and regulation of defense signaling^93^; (v) stress-inducible protein coi6.1 (log_2_FC = 8.84) associated with cold stress tolerance^94^ (vi) ras-related protein RABE1d-like (log_2_FC = 8.77) is a cellular cargo located on golgi bodies and plasma membrane^95^. In our study, DEGs such as nucleobase-ascorbate transporter 3, clathrin interactor EPSIN-2, stress-inducible protein coi6.1 and ras-related protein RABE1d-like were validated through qRT-PCR in IND99-907. Whereas, the enriched genes in Co 97010 were more related to metabolic pathways such as (i) NADH dehydrogenase subunit 6 (log_2_FC = 13.99) associated with cellular respiration and mitochondrial respiratory chain^96^; (ii) nitrate regulatory gene2 protein (log_2_FC = 9.51) regulating the nitrate transporter^97^ (iii) exocyst complex component EXO70B1 (log_2_FC = 8.65) regulating the vesicular trafficking of plasma membrane-bound kinase FLS2^98^ and (iv) Enhancer of AG-4 protein 2 (log_2_FC = 8.56) regulating the anthocyanin production pathways^99^. GO term: transport enriched genes showed that endomembrane and stress-related transport genes were enriched in IND99-907 and basic metabolic pathway related transport genes were enriched in Co 97010.

KEGG enrichment analysis identifies the statistically significant enriched pathways from a set of differentially expressed genes from RNAseq data^58^. In this study, the KEGG enrichment analysis showed the significant enrichment of MAPK signalling pathway in IND99-907 (FDR = 0.0023) as compared to non-significant expression in Co 97010 (FDR = 0.2042). The sessile plants perceive the stresses by the cascades of mitogen-activated kinase-related pathways^59, 60^ and activate the downstream regulatory genes. A total of 15 mitogen-activated kinases (MAPKs), five mitogen-activated kinase kinase (MAPKKs) and 16 mitogen-activated kinase kinase kinase (MAP3Ks) were regulating the biotic and abiotic stresses in sugarcane commercial varieties (*Saccharum spp*. hybrids)^61^. In our study, ABA signalling genes viz*.,* MAP3K1 (log_2_FC = 8.04), and protein phosphatase-2C (PP2C) (log_2_FC = 7.54) were highly enriched in IND99-907. PP2C negatively regulates the ABA signalling through inhibition and phosphorylation of SNRKs^62^. MAP3K regulates the intra- and inter-cellular genes associated with the homeostasis of reactive oxygen species^63^ and activation of ABA signalling pathways through phosphorylation of SNRK2 kinases^64^. The RAN1 copper-transporting P-type ATPase was downregulated in IND99-907, which is linked to the ethylene-response pathway^65^ and negative regulation of ethylene response enhances the plant growth^66^. Whereas in Co 97010, the late responsive genes such as MAPK (log_2_FC = 3.36), serine/threonine-protein kinase SAPK1 (log_2_FC = 8.2), and PR1 (log_2_FC = − 2.06) are significantly enriched. These genes were previously reported for their expression under abiotic stresses in many cultivated crops^67,68^.

The KEGG enrichment pathway: Biosynthesis of secondary metabolites was significantly enriched in both salt-tolerant IND99-907 (FDR = 3.56E−10) and salt-sensitive Co 97010 (FDR = 8.40E−05). Secondary metabolites are essential for physiological adaptive responses in various stimuli^69^. Secondary metabolites such as anthocyanins, flavones, and phenolics inactivate the cytotoxic ions by binding to them, thus protecting the cells from ionic oxidative damage^70^. In our study, highly expressed genes involved in the pathway biosynthesis of secondary metabolites in IND99-907 were (i) DLAT2 of pyruvate dehydrogenase complex (log_2_FC = 20.43) regulating glycolytic and citric acid pathways, and its upregulation conferring tolerance to abiotic stresses is in confirmation with previous reports^71^; (ii) glutamate decarboxylase (log_2_FC = 10.87) confer tolerance to multiple stresses by regulating the γ-amino butyrate content in plants^72^ (iii) 3-ketoacyl-CoA synthase 11-like (log_2_FC = 10.85) gene regulates the elongation of fatty acid and confer tolerance to salt stress^73^ (iv) aldose reductase (log_2_FC = 4.31) involved in biosynthesis of osmo-compatible compounds such as sorbitol^74^ (v) lysine-ketoglutarate reductase/saccharopine dehydrogenase1 (log_2_FC = 4.41) regulating the signalling of abscisic acid, jasmonate, sugar and starch starvation related pathways^75^ was enriched in IND99-907 and these genes are validated through qRT-PCR. The enriched genes in Co 97010 were also stress-related genes such as (i) acetyl-CoA acetyltransferase, cytosolic 1-like isoform X2 (log_2_FC = 8.60) associated with drought tolerance^76^; (ii) chloroplastic solanesyl-diphosphate synthase 3 (log_2_FC = 8.52) associated with prenylquinones^77^; (iii) UDP-glycosyltransferase (log_2_FC = 8.31) involved in glycosylation or inactivation of aglycones such as hormones, secondary metabolites, and its overexpression confer tolerance to abiotic stress tolerance^78^. Though there are common genes expressed in two genotypes, many genes were associated with the biosynthesis of secondary metabolites in IND99-907 as compared to Co 97010.

Many DEGs related to various metabolic pathways such as cytoskeleton organization (DHN1, DIR1 and KTN80.41), cellular membrane stabilization (EPSIN-2 and RABE1d), metabolic pathways related to biosynthesis of secondary metabolites, late embryogenesis proteins, osmolytes and sterols (PFK6-Like, SUS2, DLAT2, GAD1, LKR, SMO2-1, STE1, 7DGT, GLCAT14A, DHS, GDPDL3, HSP23.6, AKR1B1, BAN and LEA3), signal transduction (PLDZETA1, CPK15, SIP1, NCED3, PLDalpha1, ARF7, NADP-ME1, AIRP2, SNL6, ETP and SNL6), solute transport (ACA10 and PMA1), transcriptional regulation (matK, MADS26, NAC74 and MOS14), transferase or metallic homeostasis (NAAT-A) and transporters (CAT2, KCS11, CSTLP3, IRT2 and GLT) were validated through qRT-PCR in IND99-907 and Co 97010.

## Conclusion

In summary, a comparative analysis of transcriptome was carried out between two genotypes *Erianthus arundinaceus* genotype IND99-907 and salt-sensitive sugarcane genotype Co 97010 to elucidate the salt-responsive genes and metabolic pathways associated with salt-tolerance. Our findings showed clear and distinct metabolic pathways and genes involved in pathways such as MAPK signal transduction, plant hormone signalling and biosynthesis, secondary metabolite biosynthesis and hormones, biosynthesis of amino acid, transporters, and many other pathways were significantly enriched. The gene ontology enrichment analysis also revealed enrichment of many stress-related biological processes such as response to abiotic stimulus, stress, stimulus and endogenous stimulus, generation of precursor metabolites and energy, and many other biological processes. Our study has shown the metabolic pathways and genes linked with salt tolerance in IND99-907 at the transcriptome level. Further studies are required to overexpress the differentially expressed candidate genes from IND99-907 in the background of sugarcane cultivars through biotechnological approaches and development of functional markers associated with salinity tolerance.

## Materials and methods

### Plant materials and treatment

Salinity-tolerant *Erianthus arundinaceus* genotype IND99-907 collected from the saline lands from Ernakulum, India^29^ and salinity-sensitive sugarcane genotype Co 97010^30,31^ were used in this study. Single buds from each genotype with five replications were grown in pots containing river sand: red soil: farmyard manure in 1:1:1 proportion. The experiment was conducted in the polyhouse facility with a 16-h photoperiod, 30/25 °C day/night, 380 − 400 μmolm^−2^ s^−1^. Salt stress was imposed during the formative stage on the 90^th^ day after planting by irrigating with salinized water containing NaCl, CaCl_2,_ and Na_2_SO4 in the ratio of 2:2:1^30^. Salinity stress was imposed by adding 25 mM of saline water on the first day followed by incrementally adding 25 mM on every day till reaches 175 mM^100^.

### Physiological assay of IND99-907 and Co 97010 under salinity stress

The young leaf elongation was measured at an interval of 3, 6, 9, 12 and 15th days after imposing the salinity stress^19^. Samples were collected, cut into 10 cm segments and leaf fresh weight was weighed. The leaves were soaked overnight in distilled water. After soaking, excess water on the leaf surface was removed and turgid weight was quantified. The leaves were oven-dried at 37 °C and dry weight was recorded and RWC was calculated^101^. Proline was estimated by the colourimetric method from the root samples^102^.

To assess the cellular changes in the root cortical and vascular tissues, root samples of 5 cm from the root tip were sampled from the 16^th^ day of stressed and control treatments. The hand sections were made, mounted on the slide and the observed image was captured from the light microscope under 4.5X and 40X resolutions. The ultrastructure of cellular changes in selected genotypes was analysed using a Scanning Electron Microscope (FEIQuanta250, Icon analytical, FEI, USA). Root sections of 4–5 mm thick were gold coated with a sputter coater and mounted on round aluminum stubs with the aid of double side adhesive tape. The samples were scanned and selected regions were photographed. The elemental distribution of Na^+^, K^+^, Ca^2+^ and Mg^2+^ in the root sections were analysed using a line scan of EDX.

For ionic analysis in roots, the oven-dried and finely grounded roots (100 mg each) were digested with 10 ml of HNO_3_:HClO_4_ (3:1) di-acid mixture for overnight. The flasks were gently heated on a hot plate till the development of intense white fumes and the process continued till the digested mixture remains transparent. Leftover digested material made the final volume of 50 ml by adding distilled water. This solution was filtered with Whatman filter paper no. 42 and the extract were used for estimation of Na^+^, K^+^, Ca^2+^ and Mg^2+^ content by ICP-OES (ICP 9000, Shimadzu, Japan).

### Transcriptome sequencing

The gene expression patterns are differing with respect to the duration and concentration of exposure to salt stress. The isolation of short-term salt responsive gene requires few hours of exposure to salt stress and long-term salt stress gene isolation requires exposure longer duration for about a week or more. The gene expression patterns are also differing for salt shock and gradual incremental increase in salt stress. The maximum number of genes are expressed within a few hours under salt shock and after a week under gradual exposure to salt stress^100^. Hence, our study is aimed at isolation of long-term salt responsive genes under gradual exposure to salinity stress and hence, we opted incremental increase of salt stress of 25 mM till the final concentration reaches 175 mM and the total RNA from stressed and control samples of IND99-907 and Co 97010 were sampled after a week.

Total RNA was isolated from control and salt-stressed roots sampled on 10^th^ day of salinity stress from IND99-907 and Co 97010 with three independent biological replicates. Samples were prepared using Tru-Seq RNA Sample Preparation Kit v2 (Illumina Catalog No RS-122-2001) as per the manufacturer instructions. The RNA is fragmented into small pieces using divalent cations, 3’ ends were adenylated to prevent the reads from self-ligation and both 5’ and 3’ adapters were ligated. PCR amplification was carried out to generate the final cDNA libraries. The cDNA libraries of all twelve samples were quantified using Qubit and Nanodrop and validated by Agilent 2200 TapeStation System. The samples with RNA integrity number more than seven were sequenced by using Illumina *HiSeq*2500 with 2 × 100 bp in paired-end format.

### Bioinformatics analysis

Raw transcriptome sequences were quality checked using the tool FastQC (http://www.bioinformatics.babraham.ac.uk/projects/fastqc/). Poor quality reads were removed using Trimmomatic^103^ with a threshold Phred score of ≥ 30 (Q30) and a minimum length of ≥ 80 following the removal of the adapter sequences^104^. SILVA database (release 132) (https://www.arb-silva.de/) was used to remove the rRNA present in the sample by alignment using bowtie2^105^. For *de-novo* assembly, high-quality, adapter-free Q30 reads of all three biological replicates of four conditions were pooled together to generate four clustered de novo assemblies for both control and stressed samples of IND 99-907 and Co 97010 by using Trinity assembler v2.11.0 (https://github.com/trinityrnaseq/trinityrnaseq/wiki)^106^.

The quality parameters of de novo assemblies were checked by using two approaches, (i) Quast 5.0.2^107^ to obtain assembly statistics and (ii) BUSCO v4.1.2 (Benchmarking Universal Single-Copy Orthologs) analysis. Unigenes clustering was done based on 95% identity threshold using CD-HIT v4.8.1^108–110^ with parameters “cd-hit-est -c 0.95, -n 8” using the following ways, (i) for all four assemblies (containing isoforms) separately (ii) Control and Stressed unigene datasets were merged to generate a single dataset for every genotype (two unigene datasets for IND 99-907 and Co 97010) (iii) Further, unigenes clustering was performed to remove duplicate sequences to generate two reference unigene datasets for IND 99-907 and Co 97010 respectively. TransDecoder tool (https://github.com/TransDecoder/TransDecoder/wiki) was utilized to recognize the coding DNA sequences (CDS) and the long ORFs in all four unigene assemblies.

The transcript quantification by estimating the abundance of each gene was carried out using RSEM v1.2.25^111^. Differential gene expression analysis (DGE) was carried out by DESeq2 tool in R with parameters, log_2_FC > 2 (up-regulated) and log_2_FC < −2 (down-regulated) with FDR < 0.01. DESeq2 requires estimated raw counts for performing DGE, hence the raw gene counts obtained from RSEM were used for performing the same ^112,113^. Annotations were performed with databases viz*.,* NCBI-nr (1e-3) (https://www.ncbi.nlm.nih.gov/refseq/about/nonredundantproteins), Swissprot (https://www.uniprot.org/statistics/Swiss-Prot), Pfam (http://pfam.xfam.org/) and Uniprot Plants (https://www.uniprot.org/help/plants). The annotations of DEGs against NCBI-nr and Swissprot databases with an e-value of 1E-3 was done using Blast2GO v5.2.5^37^, HMMer program was used against the Pfam database and Command-line Blast was used against Uniprot Plants to retrieve their respective protein annotations.

**Figure 4:**
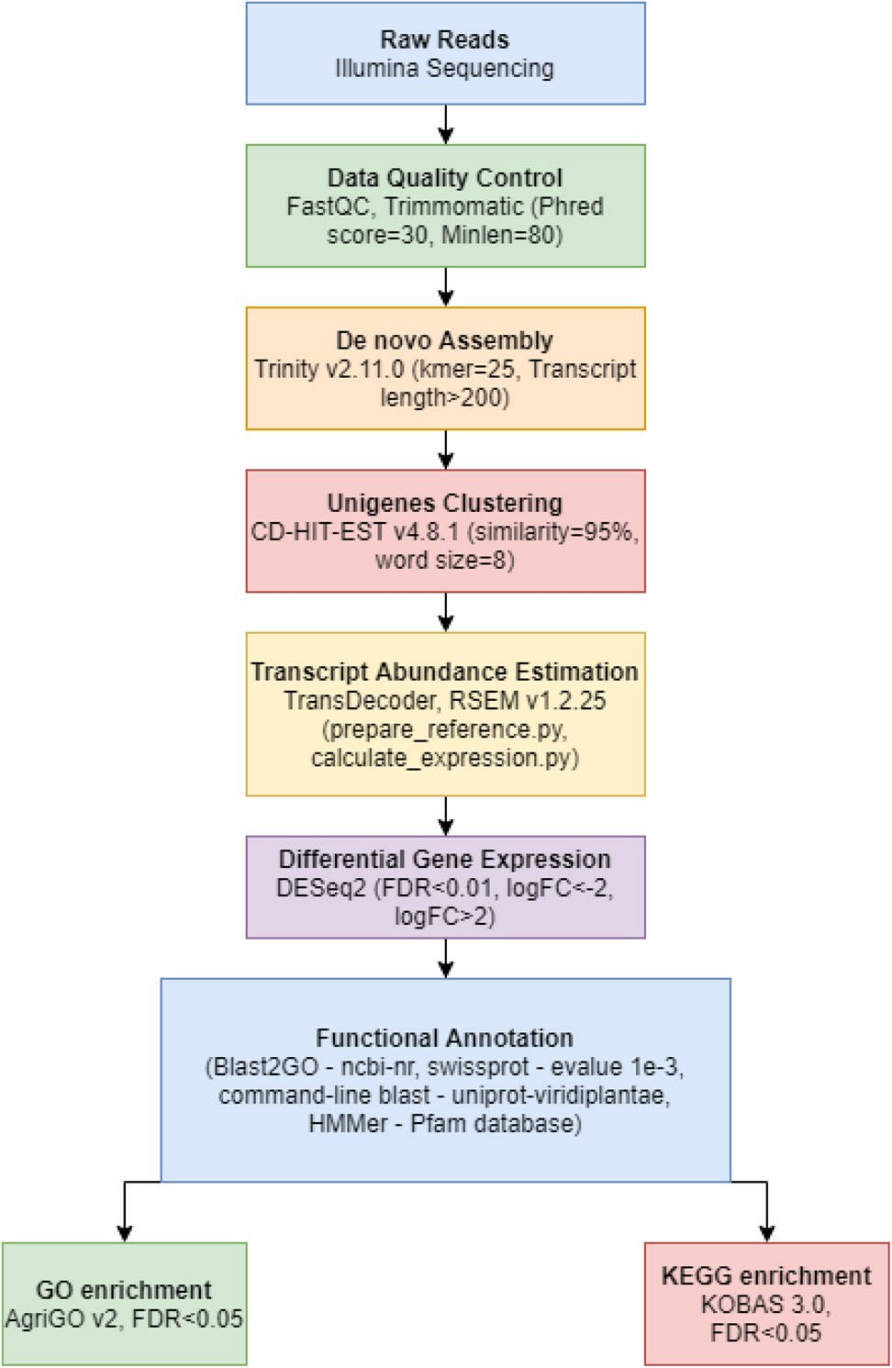
Complete Bioinformatic workflow of RNA-seq analysis involving two genotypes IND99-907 and Co 97010.

Annotation for Gene Ontology (GO mapping), InterProScan, Enzyme Code (EC) mapping was performed using Blast2GO v5.2.5^37^. GO enrichment was done using Hypergeometric test and Benjamini–Hochberg correction through AgriGO v2^79^. KOBAS 3.0 (http://kobas.cbi.pku.edu.cn/kobas3/?t=1). ^58,114–116^ was used to perform KEGG pathway enrichment. The complete workflow followed for RNASeq data analysis is given in Fig. 4.

### Validation using qRT-PCR

A total of 43 candidate DEGs were selected based on their involvement in the enriched stress-related pathways and biological processes in both stressed and control transcriptome data of IND99-907 and Co 97010 were used for validation by qRT-PCR (Supplementary Table S7). PrimerQuest tool^117^ was used to design the primers for conserved domains identified by Pfam domain search by SMART tool^118^. The total RNA was reverse transcribed by using PrimeScript 1^st^ strand cDNA synthesis Kit (Takara, Catalog No.: 6110A). The 1.0 µg of total RNA from each sample taken from stressed and control samples was used for cDNA synthesis and total cDNA was synthesized as per the manufacturer instructions. The qRT-PCR validation was carried out using StepOne Real-Time PCR System (Applied Biosystems, Canada) and each gene was validated using three biological and two technical replicates. GAPDH gene expression was used as the internal control^23^. The qRT-PCR Reaction Mix was prepared using TB Green® Premix Ex Taq™ (Tli RNase H Plus) Master Mix (Takara, Catalog No.: RR420A). The 20µL qRT-PCR mix consisted of SYBR green master mix (10µL), ROX reference dye (0.4µL), cDNA (1.0µL or 50 ng), forward and reverse primers (0.40 µl each) and molecular grade sterile water^23,119^. The initial denaturation at 95 °C for 10 min, followed by 40 cycles of denaturation at 95 °C for 15 s and, annealing and extension at 60 °C for 60 s. Relative fold changes of gene expressions were determined using the 2^−ΔΔCT^ method^120^.

### Statement of compliance

Experimental material involves the research between sugarcane wild relative *Erianthus arundinaceus* and modern sugarcane genotype (*Saccharum* spp) is in compliance with institutional, national, and international guidelines and legislation.

## Supplementary Information

Supplementary Information 1.

Supplementary Information 2.

## Data Availability

The Illumina raw reads of samples were deposited in NCBI BIO PROJECT with accession number PRJNA716503**.**

## Abbreviations

BUSCO: Benchmarking universal single-copy orthologs
DEGs: Differentially expressed genes
FDR: False discovery rate
GO: Gene ontology
SEM–EDX: Scanning electron microscopy—energy dispersive X-ray analysis
ICP-OES: Inductively coupled plasma—optical emission spectrometry
KEGG: Kyoto encyclopaedia of genes and genomes
NCBI-nr: National Center for biotechnology information-non-redundant database
Pfam: Protein family database
qRT-PCR: Quantitative real-time polymerase chain reaction
QUAST: Quality assessment tool for genome assemblies
TF: Transcription factors
