# Supplementary material for: Comparative de novo transcriptome analysis identifies salinity stress responsive genes and metabolic pathways in sugarcane and its wild relative Erianthus arundinaceus [Retzius] Jeswiet
Source: Sci Rep. 2021 Dec 31;11:24514. doi: 10.1038/s41598-021-03735-5 (PMC8720094; doi:10.1038/s41598-021-03735-5)
Supplement: Supplementary file 1 — Supplementary Information 1. [file 41598_2021_3735_MOESM1_ESM.docx]

**Comparative *de novo* transcriptome analysis identifies salinity stress responsive genes and metabolic pathways in sugarcane and its wild relative *Erianthus arundinaceus* [Retzius] Jeswiet**

Vignesh P^†1^, Mahadevaiah C^†1*^, Parimalan R^2,3^, Valarmathi R^1^, Dharshini S^1^, Nisha Singh^4,5^, Suresha GS^1^, Swathi S^1^, Mahadevaswamy HK^1^, Sreenivasa V^1^., Mohanraj K^1^., Hemaprabha G^1^, Bakshi Ram^1^, Appunu C^1*^

^1^ICAR-Sugarcane Breeding Institute, Coimbatore.

^2^ICAR-National Bureau of Plant Genetic Resources, New Delhi.

^3^Queensland Alliance for Agriculture and Food Innovation, University of Queensland, Australia

^4^ICAR-National Institute of Plant Biotechnology, New Delhi.

^5^ Institute for Genomic Diversity, Cornell University, Ithaca, NY 14853, USA

^†^ Shared co-first authors or equal contributions to the work.

^*^Corresponding authors: [C.Mahadevaiah@icar.gov.in](mailto:C.Mahadevaiah@icar.gov.in), [cappunu@gmail.com](mailto:cappunu@gmail.com),

**Supplementary Figure S1 – Gene Ontology enrichment analysis for total DEGs in IND99-907. A) GO enrichment of Biological Process (BP) for total DEGs in IND99-907 during salinity stress, B) GO enrichment analysis for cellular components in IND99-907, C) GO enrichment of Molecular Function (MF) for total DEGs in IND 99-907 during salinity stress. The density of colours from yellow to red indicates the range of statistical significance with red showing the highly significant GO terms.**


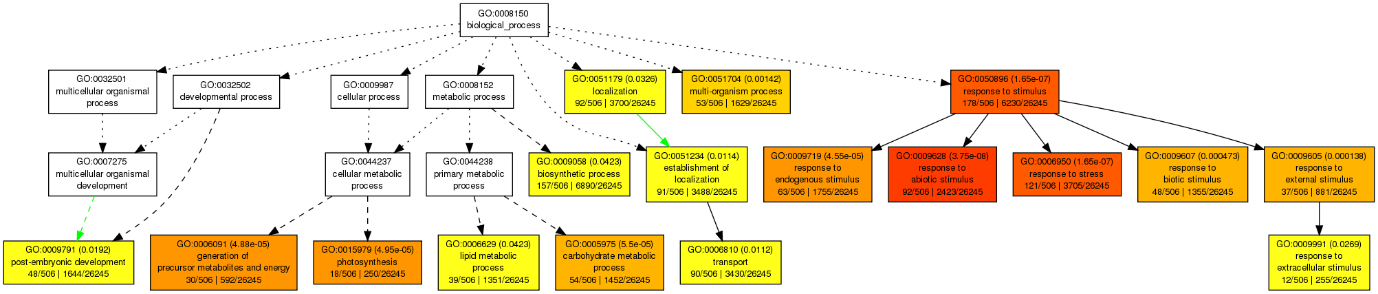


**A) GO enrichment of Biological Process (BP) for total DEGs in IND99-907 during salinity stress**


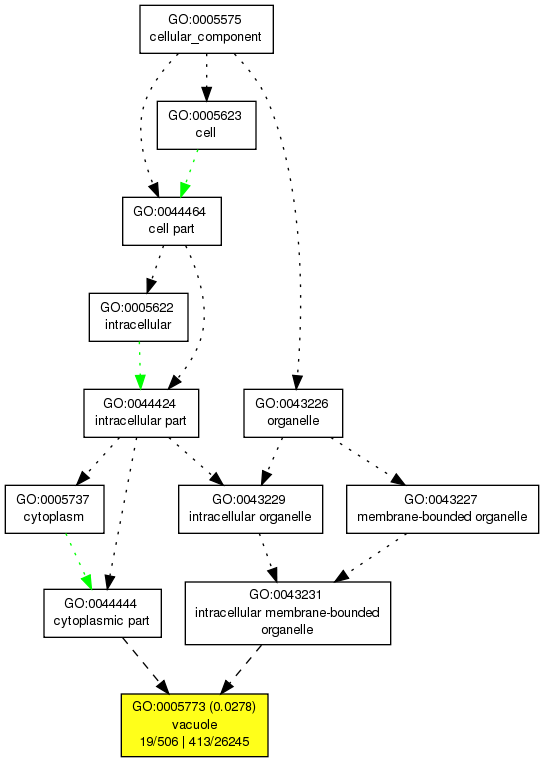


**B) GO enrichment analysis for cellular components in IND99-907**


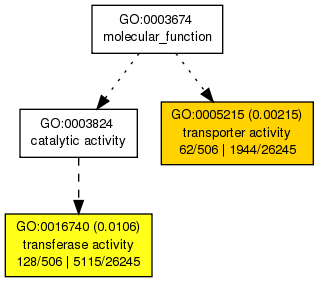


**C) GO enrichment of Molecular Function (MF) for total DEGs in IND 99-907 during salinity stress**

**Supplementary Figure S2 – A) The effect of salinity stress on plant endocytosis pathway (ko4144*) for total DEGs in IND99-907 and Co 97010. A) Endocytosis pathway in IND99-907 under salinity stress. B) Endocytosis pathway in Co 97010. Upregulated genes are marked in red colour and downregulated genes are marked in green colour.**


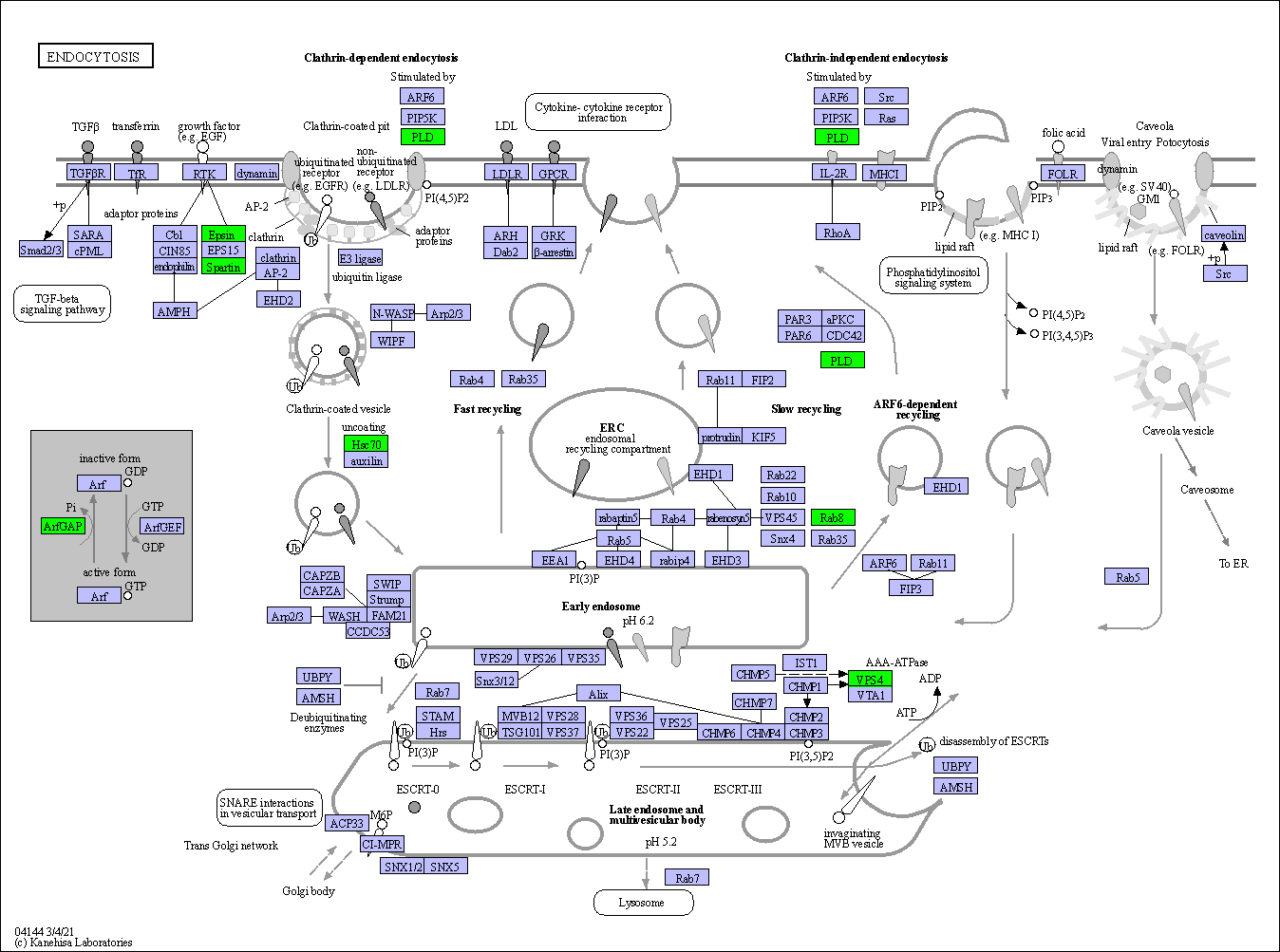

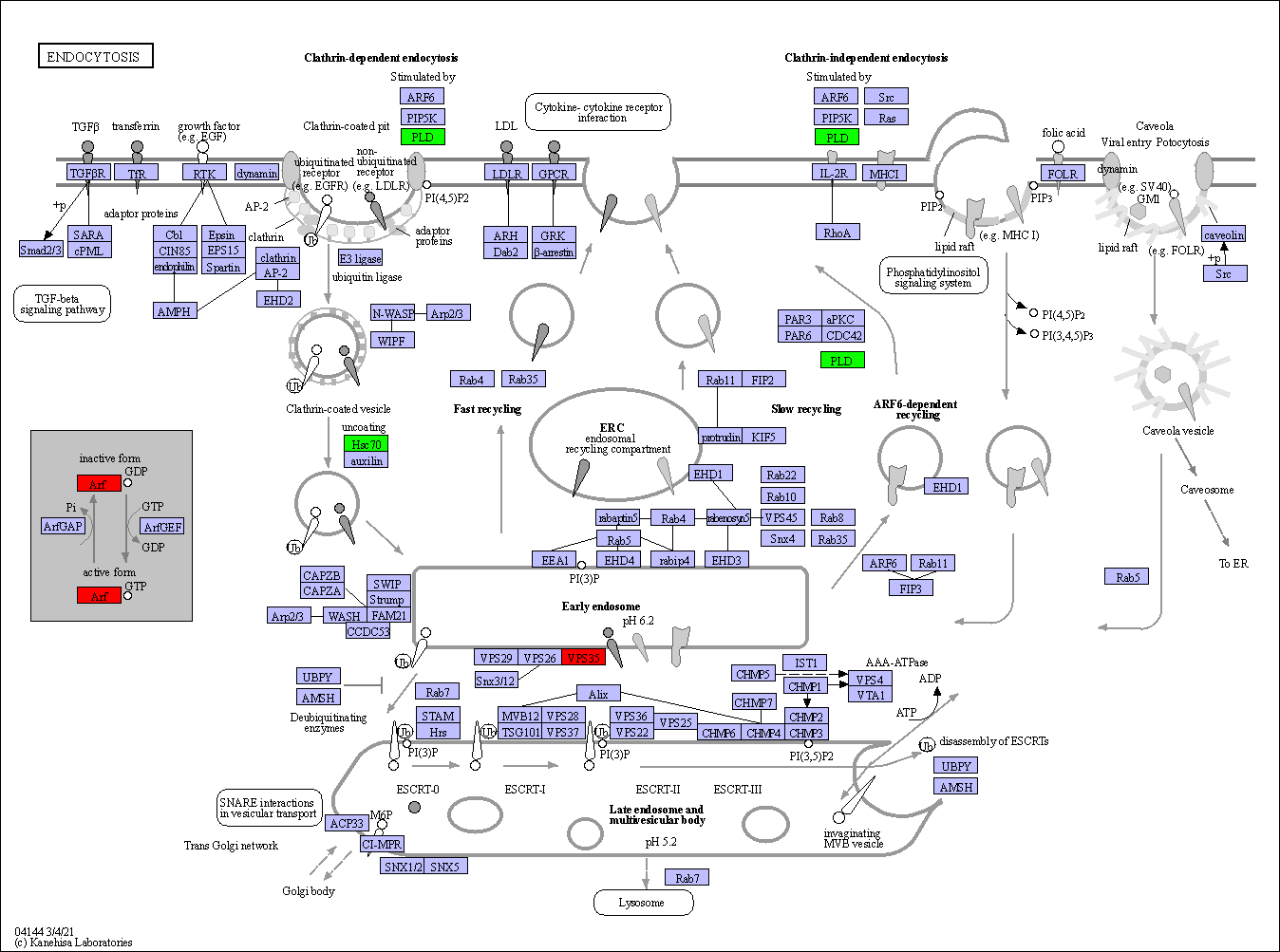


* The copyright is with Kanehisa (KEGG database project), [www.kanehisa.jp](http://www.kanehisa.jp) and Permission obtained to publish in Scientific Reports of Springer Nature Ltd in print and digital under the CC BY 4.0 open access license.

B) Endocytosis pathway in Co 97010 under salinity stress

A) Endocytosis pathway in IND99-907 under salinity stress

**Supplementary Figure S3 – A) The effect of salinity stress on oxidative phosphorylation pathway (ko00190*) for total DEGs in IND99-907 and Co 97010. A) Oxidative phosphorylation pathway in IND99-907 under salinity stress. B) Oxidative phosphorylation pathway in Co 97010. Upregulated genes are marked in red colour and downregulated genes are marked in green colour.**


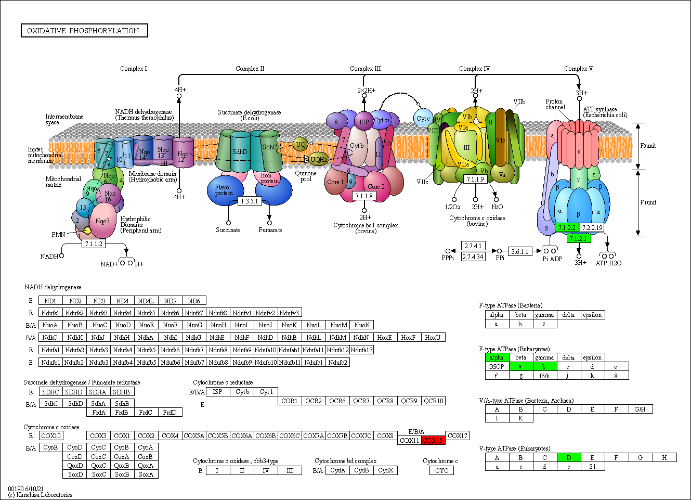

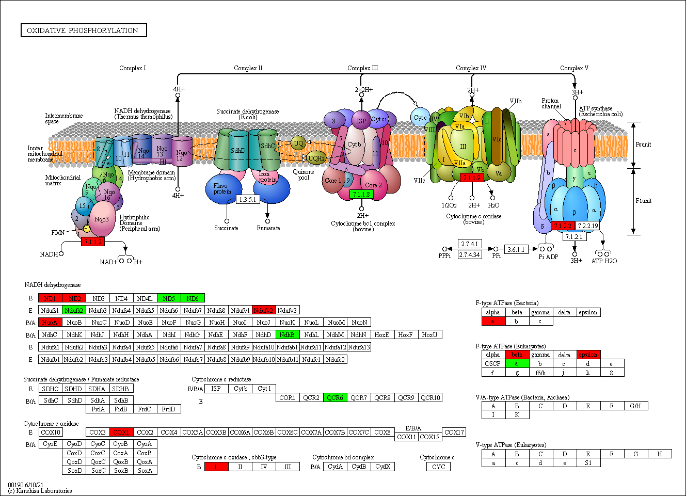


* The copyright is with Kanehisa (KEGG database project), [www.kanehisa.jp](http://www.kanehisa.jp) and Permission obtained to publish in Scientific Reports of Springer Nature Ltd in print and digital under the CC BY 4.0 open access license.

B) Oxidative phosphorylation pathway in Co 97010 under salinity stress

A) Oxidative phosphorylation pathway in IND99-907 under salinity stress
