# Supplementary material for: Comparative de novo transcriptome analysis identifies salinity stress responsive genes and metabolic pathways in sugarcane and its wild relative Erianthus arundinaceus [Retzius] Jeswiet
Source: Sci Rep. 2021 Dec 31;11:24514. doi: 10.1038/s41598-021-03735-5 (PMC8720094; doi:10.1038/s41598-021-03735-5)
Supplement: Supplementary file 2 — Supplementary Information 2. [file 41598_2021_3735_MOESM2_ESM.docx]

**Comparative *de novo* transcriptome analysis identifies salinity stress responsive genes and metabolic pathways in sugarcane and its wild relative *Erianthus arundinaceus* [Retzius] Jeswiet**

Vignesh P^†1^, Mahadevaiah C^†1*^, Parimalan R^2,3^, Valarmathi R^1^, Dharshini S^1^, Nisha Singh^4,5^, Suresha GS^1^, Swathi S^1^, Mahadevaswamy HK^1^, Sreenivasa V^1^., Mohanraj K^1^., Hemaprabha G^1^, Bakshi Ram^1^, Appunu C^1*^

^1^ICAR-Sugarcane Breeding Institute, Coimbatore.

^2^ICAR-National Bureau of Plant Genetic Resources, New Delhi.

^3^Queensland Alliance for Agriculture and Food Innovation, University of Queensland, Australia

^4^ICAR-National Institute of Plant Biotechnology, New Delhi.

^5^ Institute for Genomic Diversity, Cornell University, Ithaca, NY 14853, USA

^†^ Shared co-first authors or equal contributions to the work.

^*^Corresponding authors: [C.Mahadevaiah@icar.gov.in](mailto:C.Mahadevaiah@icar.gov.in), [cappunu@gmail.com](mailto:cappunu@gmail.com),

**Supplementary Table S1 – Summary of sequencing, pre-processing and assembly statistics data in both IND 99-907 and Co 97010**

| **Reads Summary** | **907C** | **907S** | **97010C** | **97010S** | **Combined 907 Assembly** | **Combined 97010 Assembly** |
| --- | --- | --- | --- | --- | --- | --- |
| Raw reads | 305,354,454 | 244,053,146 | 264,138,736 | 263,562,622 |  |  |
| Q30 reads | 226,446,548 | 182,093,260 | 196,117,704 | 198,198,224 |  |  |
| Q30 reads % | 74.16% | 74.61% | 74.25% | 75.20% |  |  |
| **Quast Statistics** |  |  |  |  |  |  |
| # isoforms | 205717 | 244189 | 290154 | 345745 | - | - |
| # unigenes | 179304 | 209612 | 242390 | 225911 | 315611 | 426820 |
| Contig N50 isoforms (bp) | 1506 | 1727 | 1359 | 1389 | - | - |
| Contig N50 unigenes (bp) | 1869 | 2102 | 1743 | 1722 | 2071 | 1800 |
| Largest Contig isoforms (bp) | 20317 | 23900 | 25960 | 29724 | 23900 | 29724 |
| Largest Contig unigenes (bp) | 20317 | 23900 | 25960 | 28645 | 23900 | 28645 |
| **BUSCO statistics** |  |  |  |  |  |  |
| Total BUSCOs | 425 | 425 | 425 | 425 |  |  |
| Complete BUSCOs | 297 | 340 | 313 | 314 |  |  |
| Missing BUSCOs | 49 | 31 | 44 | 52 |  |  |
| Fragmented BUSCOs | 79 | 54 | 68 | 59 |  |  |
| Completeness % | 69.8% | 80.0% | 73.6% | 73.9% |  |  |

**Supplementary Table S2- Top 30 highly differentially expressed DEGs in IND99-907 (Control vs Stress) and their corresponding fold change (log2FC) in Co 97010 (Control vs Stress) (FDR<0.01)**

| **Protein** | **log_2_FC**  **IND99-907** | **log_2_FC**  **Co 97010** |
| --- | --- | --- |
|  |  |  |
| ATP synthase CF0 subunit I | 22.66 | 0.00 |
| ATP synthase CF1 alpha subunit | 22.66 | 0.00 |
| protein LNK2 isoform X2 | 22.11 | 8.63 |
| Actin | 21.48 | 0.00 |
| abscisic stress ripening protein 2 | 21.17 | -2.76 |
| ATPase subunit 6 | 21.09 | 6.11 |
| UDP-glucose pyrophosphorylase | 20.65 | 7.96 |
| RNA polymerase beta | 20.63 | 5.28 |
| Auxin response factor 7 | 20.53 | 7.34 |
| NADP-dependent malic enzyme | 20.47 | -4.73 |
| 2,3-bisphosphoglycerate-independent phosphoglycerate mutase-like | 11.29 | 11.00 |
| late embryogenesis abundant protein 3 | 9.44 | 3.50 |
| transcription-associated protein 1-like | 9.19 | 9.94 |
| alpha/beta-Hydrolases superfamily protein | 8.50 | -7.99 |
| phospholipase D zeta 1 isoform X1 | 8.00 | 8.02 |
| CBL-interacting protein kinase 9 | 7.29 | 8.23 |
| hydroxymethylglutaryl-CoA lyase, mitochondrial | 7.24 | -7.70 |
| cleavage stimulation factor subunit 77 | 7.13 | 8.76 |
| dehydrin DHN1 | 5.81 | 6.28 |
| plant/MUD21-2 protein | 5.35 | 5.13 |
| 16.9 kDa class I heat shock protein 1 | 4.61 | 2.33 |
| galactinol synthase 2 | 3.60 | 8.00 |
| heat shock cognate 70 kDa protein | 3.56 | 3.38 |
| homeobox-leucine zipper protein HOX22 | 2.52 | 8.17 |
| vacuolar cation/proton exchanger 1a-like | 2.52 | 3.04 |
| CBL-interacting protein kinase 9 | 2.50 | 8.23 |
| GEM-like protein 5 | 2.39 | 2.46 |
| Transcription factor bHLH128 | -3.12 | 7.88 |
| ATP-dependent 6-phosphofructokinase 6-like | -3.93 | 2.46 |

**Supplementary Table S3 – The gene ontology enrichment analysis of differentially expressed genes (FDR<0.05) for salinity stress in IND99-907**

| **Sl No** | **GO ID** | | **Term** | **hits** | **P value** | **FDR**  **(0.05)** |
| --- | --- | --- | --- | --- | --- | --- |
| **A. Biological process** | | | | | | |
| 1. | GO:0009628 | | response to abiotic stimulus | 92 | 5.10E-10 | 3.80E-08 |
| 2. | GO:0006950 | | response to stress | 121 | 4.50E-09 | 1.60E-07 |
| 3. | GO:0050896 | | response to stimulus | 178 | 6.80E-09 | 1.60E-07 |
| 4. | GO:0009719 | | response to endogenous stimulus | 63 | 2.50E-06 | 4.50E-05 |
| 5. | GO:0006091 | | generation of precursor metabolites and energy | 30 | 3.30E-06 | 4.90E-05 |
| 6. | GO:0015979 | | photosynthesis | 18 | 4.10E-06 | 5.00E-05 |
| 7. | GO:0005975 | | carbohydrate metabolic process | 54 | 5.30E-06 | 5.50E-05 |
| 8. | GO:0009605 | | response to external stimulus | 38 | 1.50E-05 | 0.00014 |
| 9. | GO:0009607 | | response to biotic stimulus | 48 | 5.80E-05 | 0.00047 |
| 10. | GO:0051704 | | multi-organism process | 53 | 0.00019 | 0.0014 |
| 11. | GO:0006810 | | transport | 90 | 0.0017 | 0.011 |
| 12. | GO:0051234 | | establishment of localization | 91 | 0.0019 | 0.011 |
| 13. | GO:0009791 | | post-embryonic development | 48 | 0.0034 | 0.019 |
| 14. | GO:0009991 | | response to extracellular stimulus | 12 | 0.0052 | 0.027 |
| 15. | GO:0051179 | | localization | 92 | 0.0067 | 0.033 |
| 16. | GO:0006629 | | lipid metabolic process | 39 | 0.0095 | 0.042 |
| 17. | GO:0009058 | | biosynthetic process | 157 | 0.0098 | 0.042 |
| **B. Molecular Function** | | | | | | |
| 1. | GO:0005215 | transporter activity | | 62 | 9.40E-05 | 0.0022 |
| 2. | GO:0016740 | transferase activity | | 128 | 0.00092 | 0.011 |
| **C. cellular components** | | | | | | |
| 1. | GO:0005773 | | vacuole | 19 | 0.00065 | 0.028 |

**Supplementary Table S4 – Gene ontology enrichment analysis of differentially expressed genes (FDR<0.05) for salinity stress in in Co 97010**

| **GO Terms** | **Term** | **Hits** | **p value** | **FDR (0.05)** |
| --- | --- | --- | --- | --- |
| **A. Biological process** | | | | |
| GO:0006091 | generation of precursor metabolites and energy | 23 | 8.10E-05 | 0.0059 |
| GO:0006950 | response to stress | 81 | 0.00086 | 0.031 |

**Supplementary Table S5 – The KEGG pathways enrichment analysis (FDR<0.05) for differentially expressed genes for salinity stress in IND99-907**

| **Sl**  **No** | **KEGG Term** | **Sorghum genome ID** | **Hits** | **P-Value** | **FDR**  **(0.05)** |
| --- | --- | --- | --- | --- | --- |
| 1. | Metabolic pathways | sbi01100 | 121 | 3.43E-25 | 3.13E-23 |
| 2. | Galactose metabolism | sbi00052 | 23 | 5.37E-21 | 2.44E-19 |
| 3. | Glycolysis / Gluconeogenesis | sbi00010 | 25 | 9.84E-17 | 2.98E-15 |
| 4. | Carbon metabolism | sbi01200 | 28 | 1.27E-13 | 2.89E-12 |
| 5. | Carbon fixation in photosynthetic organisms | sbi00710 | 16 | 3.21E-12 | 5.47E-11 |
| 6. | Biosynthesis of amino acids | sbi01230 | 25 | 3.61E-12 | 5.47E-11 |
| 7. | Fructose and mannose metabolism | sbi00051 | 15 | 7.00E-12 | 9.10E-11 |
| 8. | Biosynthesis of secondary metabolites | sbi01110 | 59 | 3.13E-11 | 3.56E-10 |
| 9. | Endocytosis | sbi04144 | 19 | 3.42E-10 | 3.18E-09 |
| 10. | Lysine degradation | sbi00310 | 12 | 3.50E-10 | 3.18E-09 |
| 11. | Pyruvate metabolism | sbi00620 | 14 | 2.20E-09 | 1.82E-08 |
| 12. | Pentose phosphate pathway | sbi00030 | 9 | 1.76E-06 | 1.33E-05 |
| 13. | Plant hormone signal transduction | sbi04075 | 17 | 4.34E-06 | 3.04E-05 |
| 14. | Inositol phosphate metabolism | sbi00562 | 8 | 2.78E-05 | 0.000181 |
| 15. | Butanoate metabolism | sbi00650 | 5 | 0.000141 | 0.000854 |
| 16. | Starch and sucrose metabolism | sbi00500 | 11 | 0.000294 | 0.001671 |
| 17. | Alanine, aspartate and glutamate metabolism | sbi00250 | 6 | 0.000412 | 0.002204 |
| 18. | Protein processing in endoplasmic reticulum | sbi04141 | 12 | 0.000461 | 0.002321 |
| 19. | MAPK signaling pathway - plant | sbi04016 | 10 | 0.000485 | 0.002321 |
| 20. | Oxidative phosphorylation | sbi00190 | 9 | 0.001121 | 0.005099 |
| 21. | Plant-pathogen interaction | sbi04626 | 9 | 0.004008 | 0.017368 |
| 22. | RNA degradation | sbi03018 | 7 | 0.0052 | 0.021511 |
| 23. | Amino sugar and nucleotide sugar metabolism | sbi00520 | 8 | 0.006527 | 0.025825 |
| 24. | Spliceosome | sbi03040 | 9 | 0.01086 | 0.04008 |
| 25. | Cysteine and methionine metabolism | sbi00270 | 6 | 0.011461 | 0.04008 |
| 26. | Valine, leucine and isoleucine degradation | sbi00280 | 4 | 0.011807 | 0.04008 |
| 27. | Synthesis and degradation of ketone bodies | sbi00072 | 2 | 0.011892 | 0.04008 |

**Supplementary Table S6 – The KEGG pathways enrichment analysis (FDR<0.05) for differentially expressed genes for salinity stress in Co 97010**

| **Sl**  **No** | **KEGG Term** | **Sorghum genome ID** | **Hits** | **P-Value** | **FDR**  **(0.05)** |
| --- | --- | --- | --- | --- | --- |
| 1. | Oxidative phosphorylation | sbi00190 | 20 | 7.45E-16 | 5.59E-14 |
| 2. | Metabolic pathways | sbi01100 | 67 | 1.67E-12 | 6.28E-11 |
| 3. | Photosynthesis | sbi00195 | 8 | 2.37E-06 | 5.93E-05 |
| 4. | Biosynthesis of secondary metabolites | sbi01110 | 33 | 4.48E-06 | 8.40E-05 |
| 5. | Carbon metabolism | sbi01200 | 12 | 3.47E-05 | 0.00052 |
| 6. | Endocytosis | sbi04144 | 9 | 9.26E-05 | 0.001157 |
| 7. | Alanine, aspartate and glutamate metabolism | sbi00250 | 5 | 0.000316 | 0.003383 |
| 8. | Nitrogen metabolism | sbi00910 | 4 | 0.000684 | 0.00641 |
| 9. | Galactose metabolism | sbi00052 | 5 | 0.000946 | 0.007402 |
| 10. | Ribosome | sbi03010 | 11 | 0.000987 | 0.007402 |
| 11. | Biosynthesis of amino acids | sbi01230 | 9 | 0.001206 | 0.008225 |
| 12. | Spliceosome | sbi03040 | 8 | 0.001927 | 0.012046 |
| 13. | Valine, leucine and isoleucine degradation | sbi00280 | 4 | 0.002233 | 0.012884 |
| 14. | RNA polymerase | sbi03020 | 4 | 0.003127 | 0.01675 |
| 15. | Pentose phosphate pathway | sbi00030 | 4 | 0.003997 | 0.019984 |
| 16. | Ether lipid metabolism | sbi00565 | 3 | 0.004637 | 0.020945 |
| 17. | Synthesis and degradation of ketone bodies | sbi00072 | 2 | 0.004748 | 0.020945 |
| 18. | Carotenoid biosynthesis | sbi00906 | 3 | 0.005508 | 0.022952 |
| 19. | Protein processing in endoplasmic reticulum | sbi04141 | 7 | 0.008624 | 0.03404 |
| 20. | Carbon fixation in photosynthetic organisms | sbi00710 | 4 | 0.009927 | 0.037227 |

**Supplementary Table S7 - List of Primers used for qRT-PCR validation**

| **S**  **No** | **DEGs** | **Gene name** | **Unigene ID** | **BLAST Hits** | **Pfam Domain** | **Forward Primer** | **Reverse Primer** |
| --- | --- | --- | --- | --- | --- | --- | --- |
| 1 | DHN1 | dehydrin DHN1 | ERI-C_DN10750_c0_g1_i1.p1 | Yes | Yes | AGGGAGGAGCACAAGAC | CTTCTCCTTGATTCCCTTCTTC |
| 2 | NADP-ME1 | NADP-dependent malic enzyme | ERI-S_DN965_c0_g1_i69.p1 | Yes | Yes | GCAGCCCAAACACTACTT | CACTCCTCAATAGGAGCTTTC |
| 3 | AIRP2 | E3 ubiquitin-protein ligase AIRP2-like | ERI-S_DN2132_c0_g1_i8.p1 | Yes | Yes | GCCACGCTATGTGCATTA | GATGTCCTTGTTATCCGTGTAT |
| 4 | ARF7 | Auxin response factor 7 | ERI-S_DN1671_c0_g1_i6.p1 | Yes | Yes | ATGCCGCTGCTAACAATAG | ACCATAAAGTGCCTTCTGATAC |
| 5 | LEA3 | late embryogenesis abundant protein 3 | ERI-S_DN1898_c0_g1_i22.p1 | Yes | Yes | CACCGAGGAGAAGACTGG | TTCTGCTTGGCCTCCTC |
| 6 | NAC74 | NAC domain-containing protein 74 | ERI-S_DN392_c0_g1_i7.p1 | Yes | No | GCCACAGCAATCAAGGTAA | CACCAGAGCGGTGATAATG |
| 7 | DIR1 | dirigent-like protein | ERI-S_DN657_c0_g1_i9.p1 | Yes | Yes | GGCATCCAGTTCAAACCA | GTGGGATGTAACCCATCATC |
| 8 | PLDZETA1 | phospholipase D zeta 1 isoform X1 | ERI-S_DN2802_c0_g1_i47.p1 | Yes | Yes | CCAGGAAAGGACTACTACAATC | CCATAGAGAGCACACTGAAC |
| 9 | PFK6-like | ATP-dependent 6-phosphofructokinase 6-like | ERI-S_DN1551_c0_g1_i5.p1 | Yes | Yes | GTCAACCAGGTGTACATCATT | GTCGTTGTCGATGGTCTTG |
| 10 | SNL6 | cinnamoyl-CoA reductase-like SNL6 | ERI-C_DN1176_c1_g1_i2.p1 | Yes | No | CAAGACGATGGCGGAAA | GTAGGCAATGGATGGAGTAG |
| 11 | KTN80.4 | Katanin p80 WD40 repeat-containing subunit B1 | ERI-S_DN8743_c0_g1_i22.p1 | Yes | Yes | TTCCCGTGGTCTGTCAA | GACGAGAAGGAGTAGTCCAG |
| 12 | STE1 | Delta(7)-sterol-C5(6)-desaturase 1 | SUG-S_DN756_c0_g1_i20.p2 | Yes | Yes | CGCACTTCAGGACACATATT | GTGGCGGTAAGTTGTATGG |
| 13 | SUS2 | sucrose synthase 2 | ERI-C_DN1377_c0_g1_i34.p3 | Yes | No | CTGGAGAAGAGTGCAATCAG | CAGTACTGAGCGTCATTGAG |
| 14 | BAN | Anthocyanidin reductase | SUG-S_DN242_c0_g1_i1.p1 | Yes | Yes | GCACAGGATCCAGAGAAAG | GGCCTGATGTTGACTACTG |
| 15 | DHS | stress inducible protein coi6.1 | ERI-S_DN2053_c0_g1_i2.p2 | Yes | Yes | TTTCTGGGCTTCACTTCTAAC | GATGAGATCCTCCTCTATACCC |
| 16 | SIP1 | stress inducible protein coi6.1 | ERI-S_DN921_c0_g1_i3.p2 | Yes | No | CTTGCTTCTCTGGTAGGAATG | CGGATTCCAGGTAACAAACA |
| 17 | RABE1D | ras-related protein RABE1d-like | ERI-S_DN1918_c0_g1_i19.p2 | Yes | Yes | TTTCAAGGTTAGGACAGTTGAG | ATACAAGCAGAATGCCCATAG |
| 18 | MADS26 | MADS-box transcription factor 26 | ERI-S_DN1719_c0_g1_i2.p1 | Yes | Yes | CAGGTGACCTTCTGCAAG | GCGCGGAGAAGATGATG |
| 19 | matK | maturase K | SUG-C_DN8_c1_g1_i7.p1 | Yes | No | AACCCGTTTGGACTGATTTAT | CTAGCACATGAAAGTCGAAGTA |
| 20 | ACA10 | calcium-transporting ATPase 10, plasma membrane-type | ERI-S_DN107_c0_g1_i2.p1 | Yes | Yes | ACATCGTTCACCTCTCCA | GACAGAAGGAATGGGTTCTC |
| 21 | PMA1 | plasma membrane ATPase 1 | ERI-S_DN2505_c0_g1_i2.p1 | Yes | Yes | TCTTGTTGACTCCACGAATC | CTGGATAGGGTACATGACAATTA |
| 22 | CAT2 | Cationic amino acid transporter 2, vacuolar | ERI-C_DN2868_c0_g1_i3.p3 | yes | yes | ACATCGGCTTCCAAACAG | AGTGCTGGCAACTGAATC |
| 23 | EPSIN2 | clathrin interactor EPSIN 2 | ERI-S_DN1711_c0_g1_i13.p1 | No | No | GGCCAATCGGACTTCTTTAT | GCTGAGATGGTTGTTGATTTG |
| 24 | CPK15 | Calcium-dependent protein kinase 15 | ERI-S_DN497_c0_g1_i1.p1 | yes | yes | TTGGACTCTCCGTCTTCTT | AGCAGGATGTAGAGGATAACT |
| 25 | SIP1 | stress inducible protein coi6.1 | ERI-S_DN921_c0_g1_i3.p2 | no | no | CCGGTTGATCCTCAGTTATC | GAACTCTCTGTGACATCATCTT |
| 26 | DLAT2 | Dihydrolipoyllysine-residue acetyltransferase component 2 of pyruvate dehydrogenase complex mitochondrial | ERI-C_DN16417_c0_g1_i16.p1 | yes | yes | CTCAGTGTAACAGCTCATGG | ATTGTACCGAGTCCCTTCT |
| 27 | GAD1 | glutamate decarboxylase | ERI-S_DN184_c0_g1_i30.p1 | yes | yes | CACTGGAGAGTTTGAAGATGT | CCATTCAAGTTCAGGGTAGAG |
| 28 | KCS11 | 3-ketoacyl-CoA synthase 11-like | ERI-S_DN506_c0_g1_i8.p1 | yes | yes | GAGCTGGAGAGCAACCT | CTGTAGGCCAGCTCGTA |
| 29 | AKR1B1 | aldose reductase | ERI-S_DN5048_c1_g1_i18.p1 | yes | yes | ACCTGGATCTCTACCTTATCC | ATCCTTAACTAGCCCATCTTTC |
| 30 | LKR | Lysine-ketoglutarate reductase/saccharopine dehydrogenase1 | ERI-S_DN817_c0_g1_i119.p1 | yes | yes | TACAGGGCTACTCTTCGTTAC | CCCTTATATGTTGGGCGATTAG |
| 31 | PLDalpha1 | phospholipase D alpha 1 | ERI-C_DN805_c0_g1_i2.p1 | yes | yes | CACGCACCATCAGAAGATAG | CTTCCATCGCAAAGGTCAA |
| 32 | SMO2-1 | methylsterol monooxygenase 1-1 isoform X2 | ERI-S_DN4522_c0_g1_i1.p1 | yes | yes | GGCTTCGCCATGTCATATAG | CAATTAGGCGGATGGAGAAC |
| 33 | naat-A | Nicotianamine aminotransferase A | ERI-S_DN110_c0_g1_i3.p1 | yes | yes | AGCTCTTGCTGACAAGAATAC | GCCTCATCTGCTATGACAAATA |
| 34 | NCED3 | 9-cis-epoxycarotenoid dioxygenase 1, chloroplastic | ERI-S_DN1264_c0_g2_i5.p1 | yes | yes | GCTACAATGTCGTGTCCAA | GATGATGGCGTGGTTCTC |
| 35 | MOS14 | Transportin MOS14 | ERI-S_DN7275_c0_g1_i16.p1 | yes | yes | AGGCGCAGACAATTTGAG | CAGAAACGAAGCCAAGAAGA |
| 36 | IRT2 | fe(2+) transport protein 2 | ERI-C_DN1057_c0_g1_i4.p1 | yes | yes | CATGCTCTCGTTCCACAG | CCTCGCTCACGTCTAAATG |
| 37 | CSTLP3 | CMP-sialic acid transporter 3 | ERI-S_DN14571_c0_g2_i1.p1 | yes | yes | CAGGCTGCTCGCAATAAT | CAGCGATTACTAGAACCTTGAG |
| 38 | HSP23.6 | 23.6 kDa heat shock protein, mitochondrial | ERI-S_DN974_c0_g1_i3.p2 | yes | yes | GCTTTAGCTCGCCTGTG | GGGAGCGAAGCTTCAAAT |
| 39 | GLT | probable plastidic glucose transporter 2 | ERI-S_DN3048_c0_g1_i50.p1 | yes | yes | CCACCAGTAGCTTCACTTTAT | CAGGTGTACCAATTAGGAGTG |
| 40 | ETM | Embryogenesis transmembrane protein-like | ERI-S_DN415_c0_g1_i6.p3 | no | no | CACCGATCTTGTACTTGTAGAG | AATCAAGCGCTGGAGAAG |
| 41 | GDPDL3 | Glycerophosphodiester phosphodiesterase GDPDL3 | ERI-C_DN1124_c0_g1_i5.p1 | no | yes | GAGTCTTCACCTTCAACCTTAC | GCATTCTTGTTTCTGGGATTTC |
| 42 | GLCAT14A | beta-glucuronosyltransferase GlcAT14A-like | ERI-S_DN7775_c0_g1_i3.p2 | yes | yes | TTGCTAACACAGCTTACCC | GGTGGGTCATCCCAAATAAA |
| 43 | 7DGT | 7-deoxyloganetin glucosyltransferase | ERI-S_DN2625_c1_g2_i2.p2 | yes | yes | ACTCTGGGTGGAACTCAA | TCAATCTCCAGTCCAATTTCC |
